# Calmodulin binds and modulates K^+^-dependent Na^+^/Ca^2+^-exchanger isoform 4, NCKX4

**DOI:** 10.1074/jbc.RA120.015037

**Published:** 2020-11-23

**Authors:** Stephanie Thibodeau, Weidong Yang, Sunita Sharma, Jonathan Lytton

**Affiliations:** Department of Biochemistry & Molecular Biology, Libin Cardiovascular Institute and Hotchkiss Brain Institute, Cumming School of Medicine, University of Calgary, Calgary, Alberta, Canada

**Keywords:** membrane transport, calcium transport, sodium–calcium exchange, sodium–(calcium + potassium) exchange, plasma membrane, calmodulin (CaM), CaM, calmodulin, CaMKII, Ca^2+^-calmodulin-dependent kinase II, DAPI, 4′,6-diamidino-2-phenylindole, GST, glutathione-S-transferase, NCKX, Na^+^/(Ca^2+^+K^+^)-exchanger, N4MAb, mouse monoclonal antibody against NCKX4 obtained from NeuroMab, P12, pellet following centrifugation at 12,000*g* (crude cellular membrane fraction), P150, pellet following centrifugation at 150,000*g* (synaptic marker-enriched membrane fraction), PKC, protein kinase C, PMSF, phenylmethylsulfonyl fluoride, PSD, rabbit polyclonal serum D against NCKX4, RIPA, radioimmunoprecipitation assay buffer (1% Triton X-100, 150 mM NaCl, 25 mM TrisCl, 1 mM EDTA, pH7.5), SLC24, solute-linked carrier gene family 24, S12, supernatant following centrifugation at 12,000*g* (crude cytosolic fraction), TMS, transmembrane segment

## Abstract

The family of K^+^-dependent Na^+^/Ca^2+^-exchangers, NCKX, are important mediators of cellular Ca^2+^ efflux, particularly in neurons associated with sensory transduction. The NCKX family comprises five proteins, NCKX1–5, each being the product of a different *SLC24* gene. NCKX4 (*SLC24A4*) has been found to have a critical role in termination and adaptation of visual and olfactory signals, melanocortin-dependent satiety signaling, and the maturation of dental enamel. To explore mechanisms that might influence the temporal control of NCKX4 activity, a yeast two-hybrid system was used to search for protein interaction partners. We identified calmodulin as a partner for NCKX4 and confirmed the interaction using glutathione-S-transferase fusion pull-down. Calmodulin binding to NCKX4 was demonstrated in extracts from mouse brain and in transfected HEK293 cells. Calmodulin bound in a Ca^2+^-dependent manner to a motif present in the central cytosolic loop of NCKX4 and was abolished by the double-mutant I328D/F334D. When cotransfected in HEK293 cells, calmodulin bound to NCKX4 under basal conditions and induced a ∼2.5-fold increase in NCKX4 abundance, but did not influence either cellular location or basal activity. When purinergic stimulation of NCKX4 was examined in these cells, coexpression of wild-type calmodulin, but not a Ca^2+^ binding-deficient calmodulin mutant, suppressed NCKX4 activation in a time-dependent manner. We propose that Ca^2+^ binding to calmodulin prepositioned on NCKX4 induces a slow conformational rearrangement that interferes with purinergic stimulation of the exchanger, possibly by obscuring T331, a previously identified potential protein kinase C site.

Cellular Ca^2+^ signaling events underlie a broad range of physiological processes in all cells, particularly excitable cells such as neurons (1). In all these environments, Ca^2+^ influx must be balanced by efflux, both to terminate signals and to provide longer-term homeostasis. The family of K^+^-dependent Na^+^/Ca^2+^-exchangers, NCKX, has emerged as a particularly important pathway for modulating Ca^2+^ efflux in a variety of tissues, particularly ones associated with sensory transduction (2). There are five known members of the NCKX family, NCKX1-5, each being a product of a different *SLC24A* gene. NCKX proteins couple both Na^+^ and K^+^ gradients to the movement of Ca^2+^ with a stoichiometry of 4 Na^+^ ions in exchange for 1 K^+^ ion and 1 Ca^2+^ ion and are thus thermodynamically poised to extrude Ca^2+^ over a broad range of physiological ionic conditions (3). The NCKX proteins share an overall architecture that comprises an N-terminal hydrophobic region that is proteolytically removed during synthesis and/or trafficking, an extracellular region of variable length, a cluster of five hydrophobic transmembrane helices, a cytosolic loop of variable length, and a second cluster of five transmembrane helices (Fig. 1) (4). Among family members, the highest level of amino acid conservation is within the transmembrane regions, particularly those denoted the “α-repeats,” while the lowest similarity is found in the large hydrophilic loops, extracellularly near the N-terminus, and cytosolically near the protein center (5).

**Figure 1:**
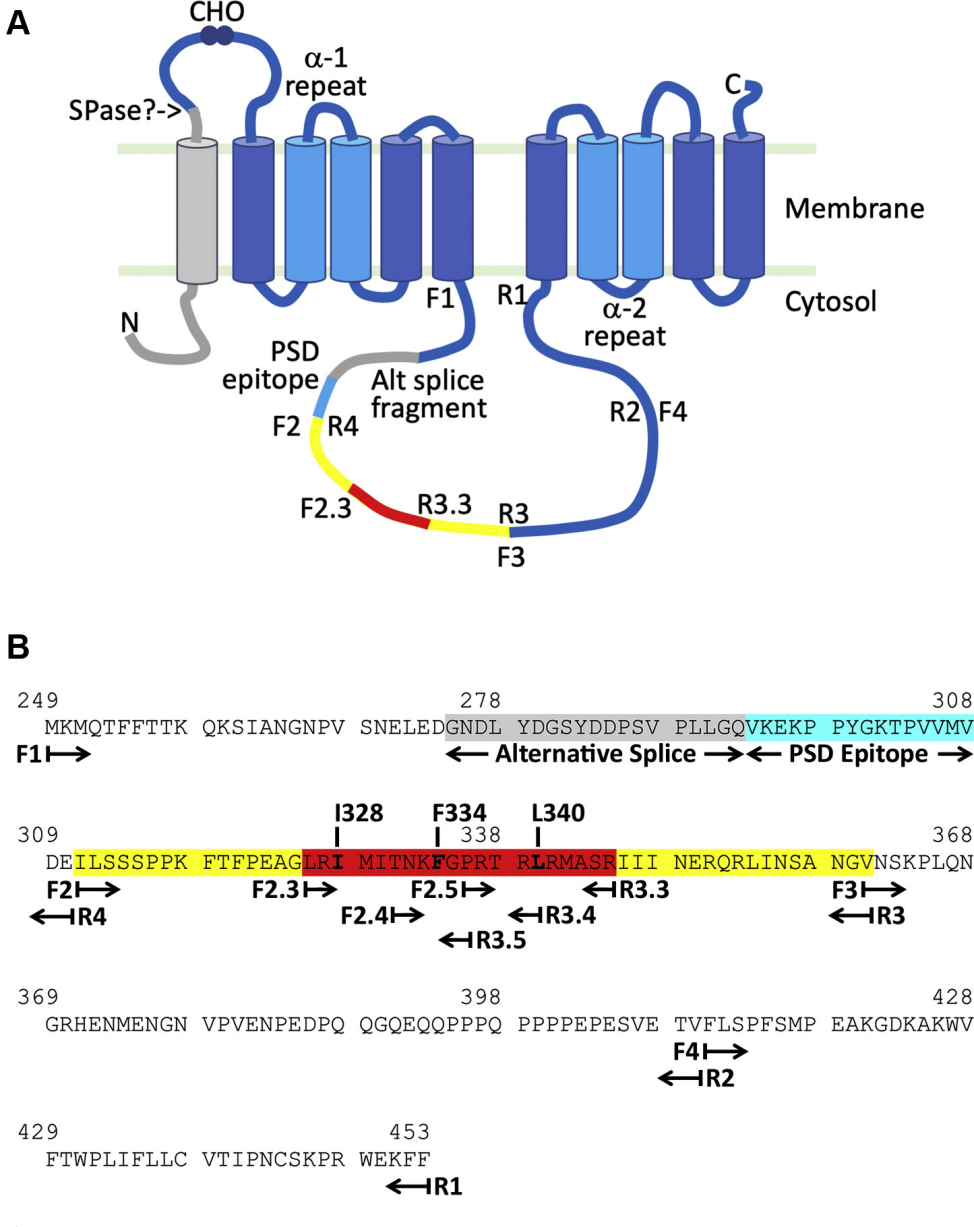
**Schematic representation of the NCKX4 protein and its cytoplasmic loop.***A*, a cartoon model for the anticipated membrane topology of the NCKX4 protein, illustrating the N-terminal hydrophobic transmembrane span (in *gray*) thought to be proteolytically processed and removed during synthesis or trafficking to the plasma membrane (*SPase?*, the putative cleave site), a glycosylated (*CHO*) extracellular loop, the two clusters of five transmembrane spans, which each include the highly conserved *α-1* and *α-2* repeat regions with opposite orientation, and between them the large central cytosolic loop. *Alt splice fragment*, the location of a 19 amino acid segment encoded by an alternatively spliced exon (*gray*); *PSD epitope*, the 15 amino acid region used as an antigen to raise the rabbit anti-NCKX4 polyclonal serum D (*cyan*); *F1*, *F2*, *F2.3*, *F3*, *F4*, *R1*, *R2*, *R3*, *R3.3*, *R4*, the sites of primers used to generate fragments of the cytosolic loop. The F2R3 segment is highlighted in *yellow*, and the F2.3R3.3 segment is highlighted in *red*. *B*, the sequence of the mouse NCKX4 cytosolic loop in single-letter amino acid code and numbered according to coordinates of the full length protein including the segment encoded by the alternatively spliced exon (*gray*). The position of the PSD epitope (*cyan*), the location of the various PCR primers, the F2R3 (*yellow*) and F2.3R3.3 (*red*) regions, as well as three amino acids involved in CaM binding, are all highlighted. Note that for all studies in this report, cloned fragments of mouse NCKX4 excluded the alternatively spliced region, although amino acid numbering has been used according to the longer protein shown in this figure.

NCKX proteins function in a variety of cells and tissues, influencing a range of physiological functions. NCKX1 was the first family member to be described and characterized for its essential role in Ca^2+^ homeostasis underlying visual adaptation in rod photoreceptor outer segments (6). NCKX2 was identified next as a significant contributor to Ca^2+^ flux in central nervous system neurons and important for normal synaptic plasticity at hippocampal Schaffer collateral-CA1 synapses (7, 8). In short order, NCKX3 and NCKX4 were also described, based largely on DNA sequence database entries, and shown to be expressed in the brain as well as distributed more broadly in other tissues (9, 10). Subsequently, NCKX4 together with NCKX2 was shown to be critical for normal signal termination and adaptation in cone photoreceptors (11). NCKX4 was also demonstrated to have additional roles in sensory termination and adaptation in olfactory neurons (12), in melanocortin-dependent satiety signaling centered in the paraventricular nucleus of the hypothalamus (13), and in dental enamel deposition (14, 15). NCKX5, identified as the gene underlying the zebrafish hypopigmentation mutant called *Golden*, was also shown to have a quantitatively important role in skin pigmentation in humans (16). NCKX5 is located within melanocytes, in vesicles associated with the trans-Golgi (17). This is in contrast to NCKX1 and NCKX2, for which there is strong evidence for cell surface expression (18). Although NCKX3 and NCKX4 activity can be measured at the plasma membrane when expressed in heterologous systems (9, 10), their endogenous cellular location has not been definitively determined.

The role of NCKX isoforms in adaptation and termination of sensory signals, as well as in synaptic plasticity, suggests that temporal modulation of activity might be important for tuning their contribution to physiological regulation (2). Indeed, in rod photoreceptors, NCKX1 is thought to interact with the resident cyclic nucleotide-gated channel, creating a cooperatively functioning protein complex, which responds to different light illumination levels (19, 20). Similar regulatory interactions have been proposed for NCKX2 in cone photoreceptors (20). Studies have also established that neuronal NCKX2 can be stimulated by protein kinase C (PKC) (21). Furthermore, both NCKX2 and NCKX4 activity can be stimulated by purinergic activation, which may be initiated by ATP released as a signaling comodulator together with neurotransmitters from synaptic and/or secreted vesicles (22). For NCKX4, purinergic stimulation has been shown to require activation of both PKC and Ca^2+^/calmodulin-dependent kinase II (CaMKII) (22), while PKC activation alone is sufficient for purinergic stimulation of NCKX2 (21).

To deepen our understanding and to illuminate mechanistic details underlying the regulation of NCKX proteins, particularly the NCKX4 isoform, we employed a yeast two-hybrid system to investigate and identify interaction partners for NCKX4. Here, we describe calmodulin (CaM) as a critical interaction partner for NCKX4 with important consequences for regulation by purinergic signaling.

## Results

### Yeast two-hybrid screen

To search for putative interaction partners for NCKX4, we chose the yeast two-hybrid approach (23, 24) implemented *via* the Dualhybrid kit from Dualsystems Biotech. Bait constructs made in the pLexA vector consisted of the entire central cytosolic loop of mouse NCKX4 (Fig. 1), lacking the region subject to alternative splicing because this is the major isoform expressed in the brain (10, 22), fused either N-terminal or C-terminal to the LexA DNA-binding domain. Both N-terminal and C-terminal constructs were then used to screen a normalized mouse brain library constructed in the pGAD (Gal4 activation domain) prey vector. A screen of about 10^6^ clones with each bait resulted in an aggregate of 15 positive colonies. These clones were isolated and independently retested for activation by the NCKX4 bait, after which four clones remained positive, all isolated using the NCKX4 construct in the pLexA-C vector. Sequencing revealed that these four isolates corresponded to three different clones, one identified twice, from the CaM genes, Calm2 or Calm3. In all cases, the entire CaM open reading frame was included, encoding identical 149 amino acid proteins.

**Figure 2:**
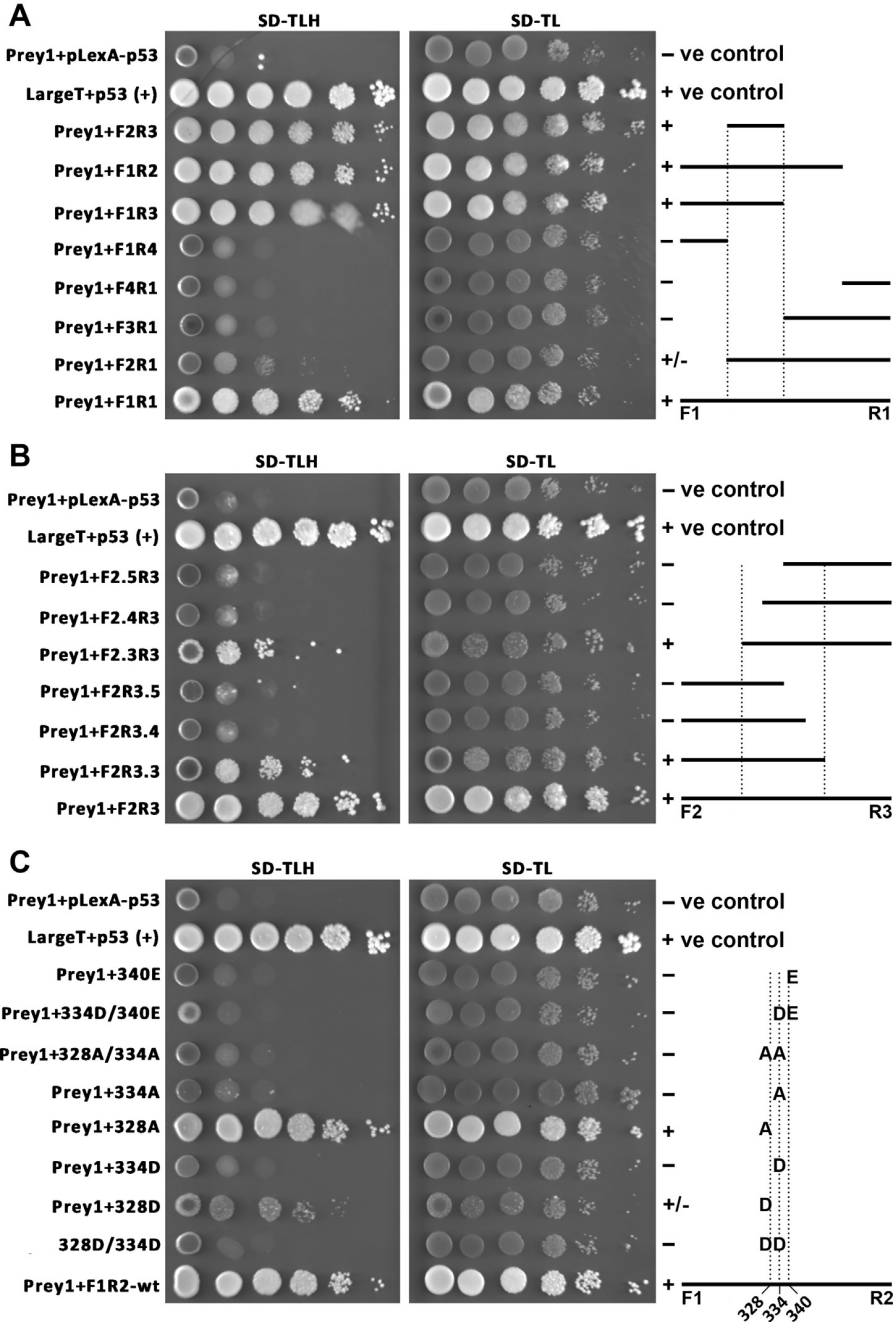
**Yeast two-hybrid analysis of the NCKX4–CaM interaction.** A yeast cell growth assay was used to define the segments and amino acids necessary for the NCKX4–CaM interaction. *Prey1* is the original clone 1 identified by the yeast two-hybrid screen, and encodes full-length mouse Calm2 in the pGAD activation vector. Control constructs correspond to the pLexA-N-p53 bait and the pACT-largeT activation vectors. Mouse NCKX4 fragments corresponding to segments defined by the indicated F and R PCR primers in the pLexA-C bait vector were transformed together with the Prey1 activation construct, into NMY51 yeast cells, plated at serial tenfold dilutions, left to right, and grown on either SD-TL (non-selective) or SD-TLH (selective) media as shown. Growth of diluted strains under selective conditions is indicative of an interaction. Schematics of the NCKX4 loop fragments tested are shown at right. Interactions were scored +, − or ±, corresponding to growth, no growth, or limited growth. *A*, the NCKX4 cytoplasmic loop, F1R1, and the various fragments defined by different F or R PCR primers were tested. *B*, the NCKX4 cytoplasmic loop fragment, F2R3, and various subfragments defined by different F or R PCR primers were tested. *C*, the NCKX4 cytoplasmic loop fragment, F1R2, and F1R2 fragments containing the indicated mutations were tested. See Figure 1 for the locations of primers and mutations. The illustrated data are representative of at least four independent determinations.

To localize the site of interaction with CaM on the NCKX4 intracellular loop, the original full-length bait was subdivided into four fragments using different PCR primers (Fig. 1), and the interaction of these fragments with the confirmed CaM prey, clone 1, was tested using a growth assay. As illustrated in Figure 2*A*, strong growth on selective plates indicated that the full-length NCKX4 loop fragment (denoted F1R1) and fragments sequentially truncated at the C-terminal end, F1R2 and F1R3, but not F1R4, were positive for interaction with the CaM. Similar truncations from the N-terminal end indicated a weak interaction for F2R1 and no interactions for F3R1 or F4R1 fragments. These results implicated the F2R3 region as the CaM-binding site, and a test of this fragment was positive for interaction. The F2R3 fragment was then further subdivided using PCR primers (Fig. 1), and positive interactions were observed with fragments F2.3R3 and F2R3.3, but not the shorter ones (Fig. 2*B*). These data defined the region Leu-326–Arg-345 (amino acids LRIMITNKFGPRTRLRMASR; Fig. 1) as the CaM interaction site. Indeed, this region displays the amphipathic, hydrophobic, and basic characteristics expected for a classical CaM-binding site (25). Key hydrophobic residues within the context of the F1R2 fragment were tested for their role in the CaM interaction by mutation (Fig. 2*C*). Mutation of Ile-328 to Ala (I328A) was without effect, while mutation to Asp (I328D) had a modest effect on the interaction. Mutation of Phe-334 to either Ala (F334A) or Asp (F334D) prevented the interaction, as did mutation of Leu-340 to Glu (L340E). Double mutations F334D/I340E, I328A/F334A, and I328D/F334D also abolished interaction with CaM.

### Glutathione-S-transferase fusion protein-binding tests

**Figure 3:**
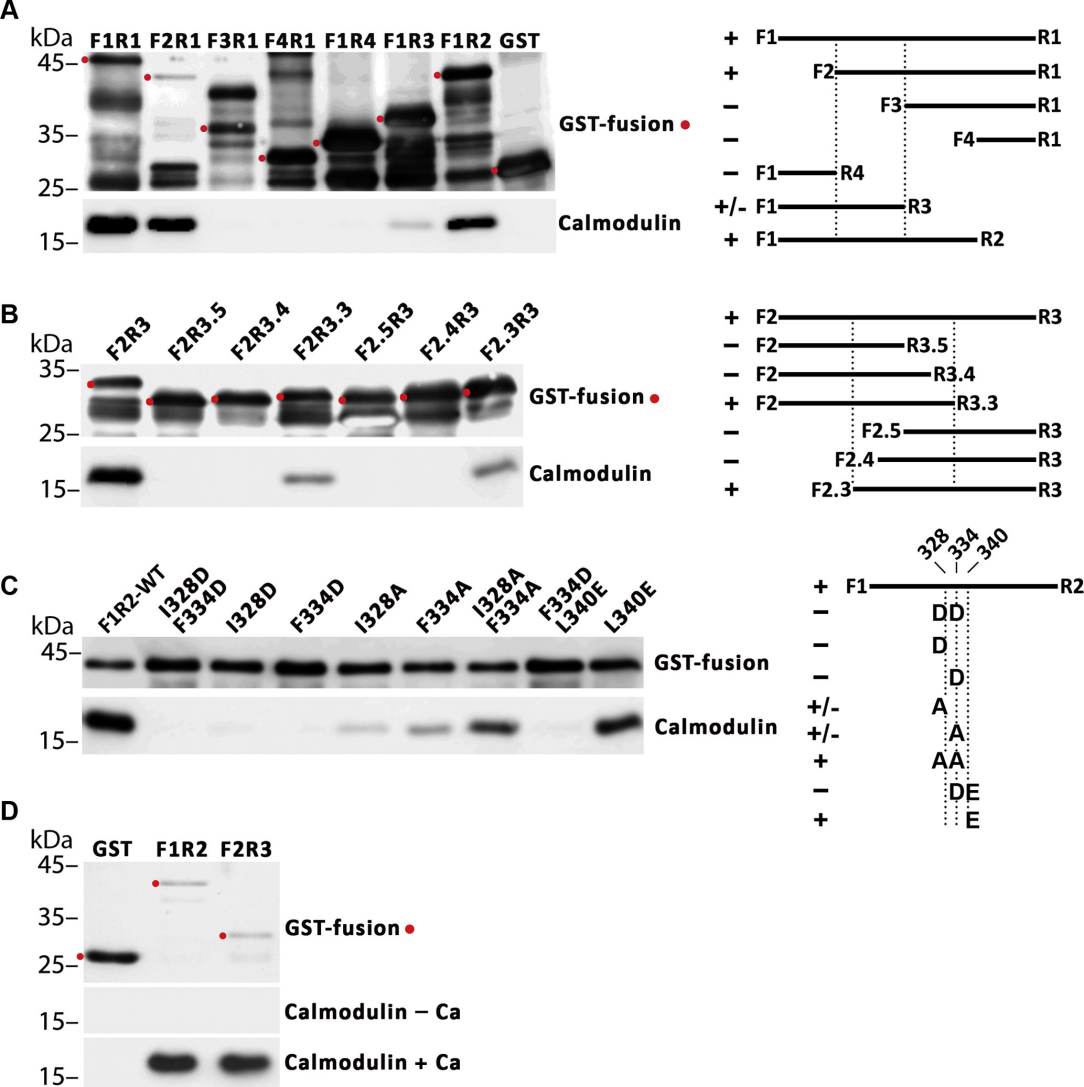
**GST-binding analysis of the NCKX4–CaM interaction.** GST fusion proteins encoding different regions of the mouse NCKX4 intracellular loop defined by the indicated F and R PCR primers were prepared in *E. coli* and immobilized on Glutathione Sepharose beads. The beads were incubated with CaM in the presence of 0.1 mM CaCl_2_, washed extensively and then eluted in sample buffer, split into two equal aliquots, and analyzed separately for GST and CaM by SDS-PAGE and immunoblot. The position of molecular mass markers (in kDa) is indicated on the left side of each gel. The full-length GST fusion protein in each sample is indicated with a *red dot*. Schematics of the NCKX4 loop fragments tested are shown at right. Interactions were scored +, − or ±, corresponding to strong, no, or weak interaction. *A*, the NCKX4 cytoplasmic loop, F1R1, and various fragments defined by the indicated F or R PCR primers were tested for interaction. *B*, the NCKX4 cytoplasmic loop fragment, F2R3, and various subfragments defined by the indicated F or R PCR primers were tested for interaction. *C*, the NCKX4 cytoplasmic loop fragment, F1R2, and F1R2 fragments containing the indicated mutations were tested for interaction. *D*, Ca^2+^ requirement for binding of CaM to NCKX4 loop fragments F1R2 or F2R3. CaM binding in either the presence or absence of 0.1 mM CaCl_2_ to the GST fusion proteins immobilized on Glutathione Sepharose was tested. See Figure 1 for the locations of primers and mutations. The illustrated data are representative of at least four independent determinations.

Next, we tested if the NCKX4–CaM functional interactions identified by yeast two-hybrid corresponded to direct physical protein interactions using purified components. For this, the same NCKX4 cytosolic loop fragments utilized in the yeast two-hybrid assays were moved into the glutathione-S-transferase (GST) fusion protein vector, pGEX-4T-1 (26, 27). The induced GST fusion proteins were immobilized on Glutathione Sepharose beads and tested for their ability to bind purified CaM (Fig. 3). As predicted by the yeast two-hybrid tests and illustrated in Figure 3*A*, CaM bound to fragments F1R1, F1R2, more weakly to F1R3, and not to F1R4 (sequential C-terminal truncations). F2R1 also bound CaM, but not F3R1 or F4R1 (sequential N-terminal truncations). CaM binding to fragment F2R3 was then confirmed and the location of CaM binding further restricted using sequential N- and C-terminal deletions. Binding was observed for fragments F2R3.3 and F2.3R3, but not for fragments F2R3.5, F2R3.4, F2.5R3, or F2.4R3 (Fig. 3*B*), thereby restricting the CaM-binding region to F2.3R3.3, corresponding to amino acids 326-LRIMITNKFGPRTRLRMASR-345 (Fig. 1). This result therefore confirmed using direct binding the same CaM-binding region identified genetically with the yeast two-hybrid assay. We next tested the influence of individual amino acid mutations on CaM binding (Fig. 3*C*). The interaction was lost with I328D, F334D, or the double mutants, I328D/F334D and F334D/L340E; substantially reduced with I328A or F334A; but only modestly reduced with the double-mutant I328A/F334A or with L340E. The effect of individual mutations on *in vitro* binding to GST was not identical to the effect on functional interaction using yeast two-hybrid (Fig. 2*C*), which may reflect the different conditions used in the two assays as well as the fact that the fusion proteins were oriented with the NCKX4 fragments at the N-terminus for yeast two-hybrid, but at the C-terminus for GST binding. Nevertheless, it is clear that the I328D and F334D mutations, and particularly the combined double mutation, were particularly effective at eliminating both physical and functional interaction between CaM and the NCKX4 loop fragment. Finally, we determined that the interaction between CaM and the NCKX4 loop fragment depended upon the presence of Ca^2+^ (Fig. 3*D*). Thus, the functional interaction between CaM and the NCKX4 loop region L326–R345 observed with the yeast two-hybrid assay was also confirmed using a direct *in vitro* GST fusion protein-binding assay.

### Interaction between brain NCKX4 and calmodulin

**Figure 4:**
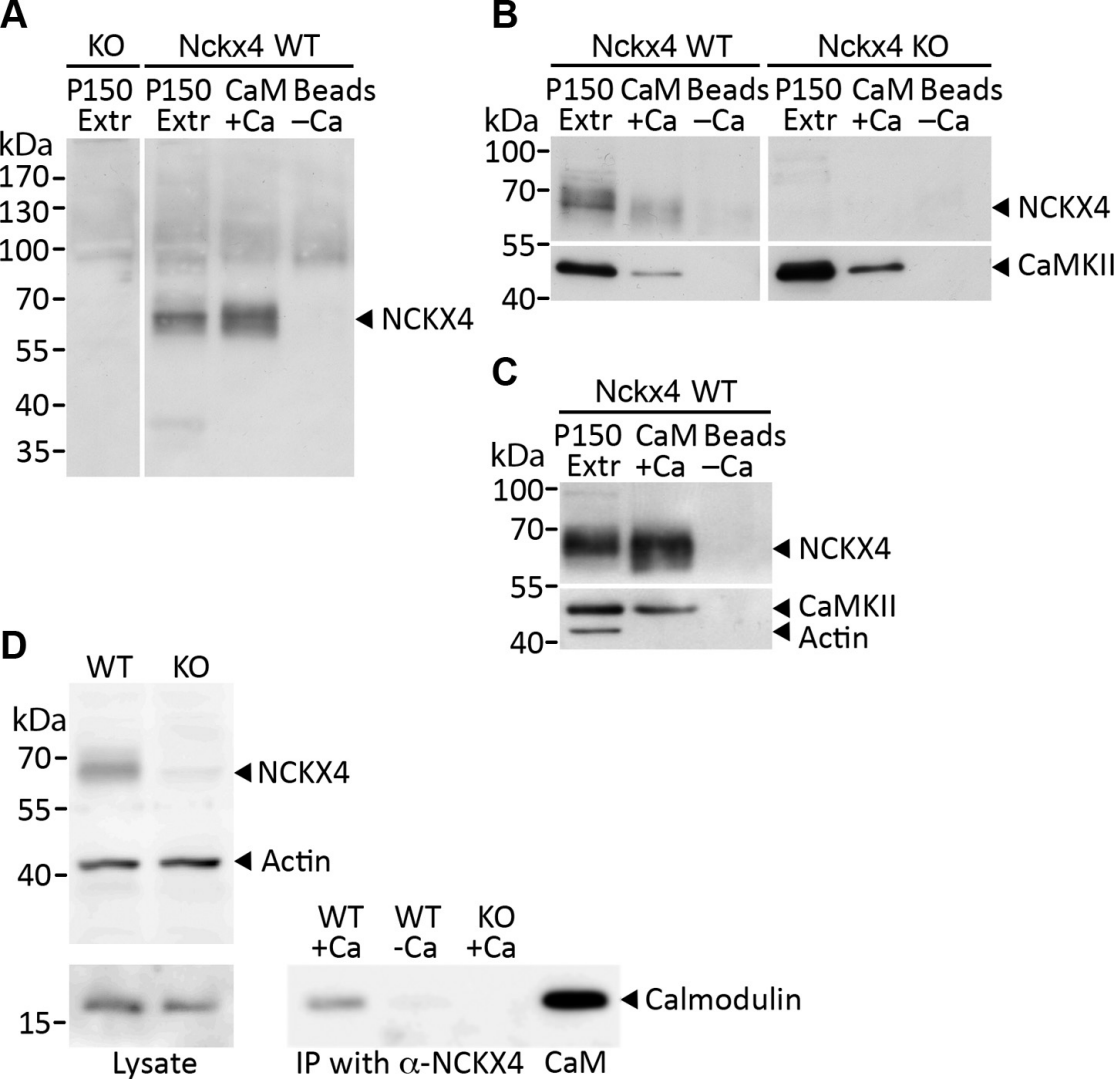
**CaM binds to NCKX4 in extracts from mouse brain.***A*, *B*, and *C*, 200 μg of mouse brain membranes (P150) from *Nckx4* wild-type (*WT*) or knockout (*KO*) mice were solubilized in 1 ml RIPA buffer and incubated with CaM affinity resin beads in the presence (+) or absence (−) of Ca^2+^. The beads were washed and eluted with sample buffer. Two microgram samples of the P150 extract (*Extr*) and 10 μl out of 15 μl of eluate from the beads (*Beads*) were analyzed by SDS-PAGE and immunoblot for NCKX4, CaMKII, and actin using separated strips of membrane from the same gel. NCKX4 and CaMKII, but not actin, bound to the CaM affinity resin beads in a Ca^2+^-dependent manner. *D*, 200 μg of solubilized P150 membrane fraction from wild-type or *Nckx4* knockout mice was supplemented with 10 μg CaM in the presence or absence of Ca^2+^, and NCKX4 was immunoprecipitated with PSD antibody. Two microgram samples of solubilized sample (Lysate) and 10 μl out of 15 μl of the immunoprecipitated samples were analyzed by immunoblot for the presence of CaM. Purified CaM (2 ng) was added to a separate lane as a size marker, where noted. The position of molecular mass markers (in kDa) is indicated on the left side of each gel. These images are representative of three independent experiments.

Having established using both genetic and biochemical *in vitro* methods that CaM interacts with a segment in the central cytosolic loop of NCKX4, we next investigated if CaM was bound to the intact NCKX4 protein in mouse brain. NCKX4 was found preferentially in a brain homogenate membrane fraction enriched in synaptic markers (13, 28) as a diffuse band of around 65kDa, absent from animals in which the *Nckx4* gene has been knocked out (Fig. 4*A*). When a detergent extract of this fraction was passed over CaM immobilized on Sepharose beads, a band corresponding to NCKX4 was retained, in a Ca^2+^-dependent manner, which was not observed in the sample from the *Nckx4* knockout, thereby demonstrating specificity (Fig. 4, *A*–*C*). As a positive control, we demonstrated that CaMKII, a known CaM-binding protein also present in the synaptic marker-enriched membranes, was also retained on CaM beads in a Ca^2+^-dependent manner (Fig. 4, *B*–*C*). Actin, also associated with this membrane fraction, did not bind to the CaM beads, reinforcing the specificity of NCKX4–CaM interaction (Fig. 4*C*).

The synaptic marker-enriched brain fraction, in which NCKX4 was found, contained very little endogenous CaM, presumably since Ca^2+^ was chelated with EDTA during preparation of this fraction. Therefore, to investigate the potential for a NCKX4–CaM interaction in solution, purified CaM was added back to a detergent extract of the synaptosome-enriched fraction either with or without Ca^2+^ present, and NCKX4 was immunoprecipitated. As shown in Figure 4*D*, CaM was recovered from the NCKX4 immunoprecipitate in a Ca^2+^-dependent manner, but was absent when a membrane fraction from *Nckx4* knockout animals was used.

### Analysis of the NCKX4–calmodulin interaction in HEK293 cells

**Figure 5:**
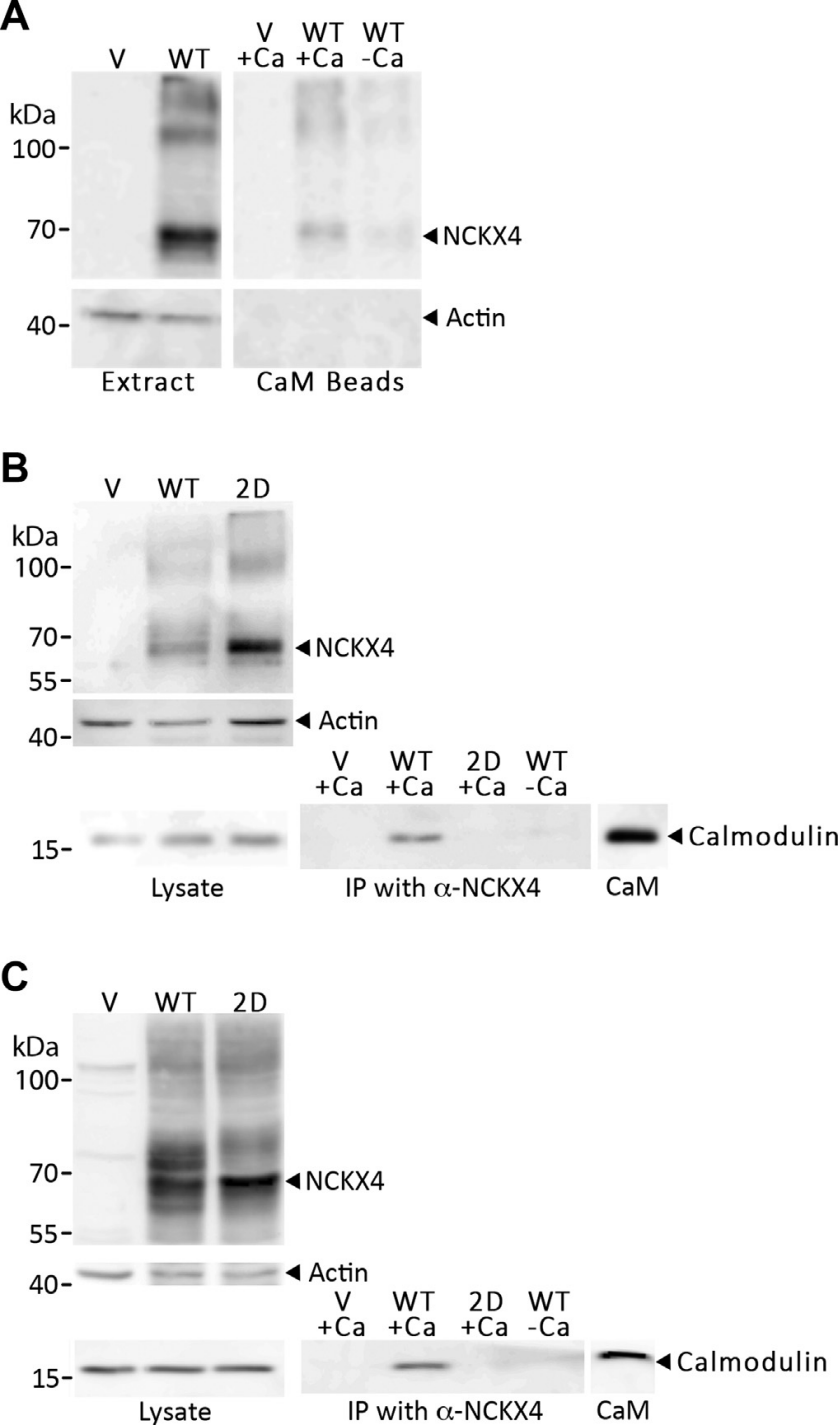
**CaM binds to NCKX4, but not the NCKX4 I328D/F334D double mutant, in extracts from transfected HEK293 cells.***A*, HEK293 cells were transfected with cDNA constructs corresponding to vector-only negative control (*V*) or wild-type mouse NCKX4 tagged at the C-terminal end with a triple-HA epitope (*WT*). Approximately 20 μg of an enriched membrane fraction (P12) was solubilized in RIPA buffer with ovalbumin, incubated with CaM affinity resin beads in the presence or absence of Ca^2+^, and eluted in sample buffer. Three micrograms of solubilized membrane samples (*Extract*) and 10 μl out of 15 μl eluate (*CaM Beads*) were analyzed by SDS-PAGE and immunoblot for actin and for NCKX4 using an anti-HA antibody. Actin was used both as a loading control for the solubilized sample and as a negative control for the CaM pulldown. *B*, HEK293 cells were transfected with cDNA constructs corresponding to vector-only negative control (*V*), wild-type mouse NCKX4 (*WT*) or mouse NCKX4 containing the I328D/F334D double mutant (*2D*). Cells were homogenized and fractionated. Approximately 20 μg of P12 membrane fraction and 100 μg of S12 fraction were combined, solubilized in 1 ml of RIPA buffer, and immunoprecipitated with anti-NCKX4 PSD antibody in the presence or absence of Ca^2+^. Two microgram samples of P12 and S12 (*Lysate*) and 10 μl out of 15 μl of the immunoprecipitated samples (*IP*) were analyzed by SDS-PAGE and immunoblot with antibodies against NCKX4 (PSD), actin (as a loading control), and CaM. Purified CaM (2 ng) was added to a separate lane as a size marker, where noted. *C*, HEK cells were transfected as described for panel B, solubilized directly in RIPA buffer, and approximately 500 μg used for immunoprecipitation as described above. Lysate lanes contain 20 μg samples of each extract. The position of molecular mass markers (in kDa) is indicated on the left side of each gel. These images are representative of four independent experiments.

We have previously used recombinant expression in HEK293 cells as a system to assess expression, function, and regulation of NCKX proteins (10, 22, 29). Therefore, full-length wild-type NCKX4, or full-length NCKX4 into which both mutations, I328D and F334D, were introduced (which prevented CaM binding to GST fusion fragments), was expressed in HEK293 cells. As illustrated in Figure 5*A*, wild-type NCKX4 was captured on CaM beads in a Ca^2+^-dependent manner. Figure 5, *B*–*C* demonstrated that wild-type NCKX4, but not the I328D/F334D double-mutant NCKX4, brought down endogenous CaM in a Ca^2+^-dependent manner from solubilized lysates of transfected cells using a coimmunoprecipitation assay. In the experiment shown in panel B of Figure 5, separated membrane and soluble fractions from homogenized cells were combined together prior to solubilization and immunoprecipitation. Essentially identical results were obtained when transfected cells were solubilized and directly subjected to coimmunoprecipitation (panel C of Fig. 5).

**Figure 6:**
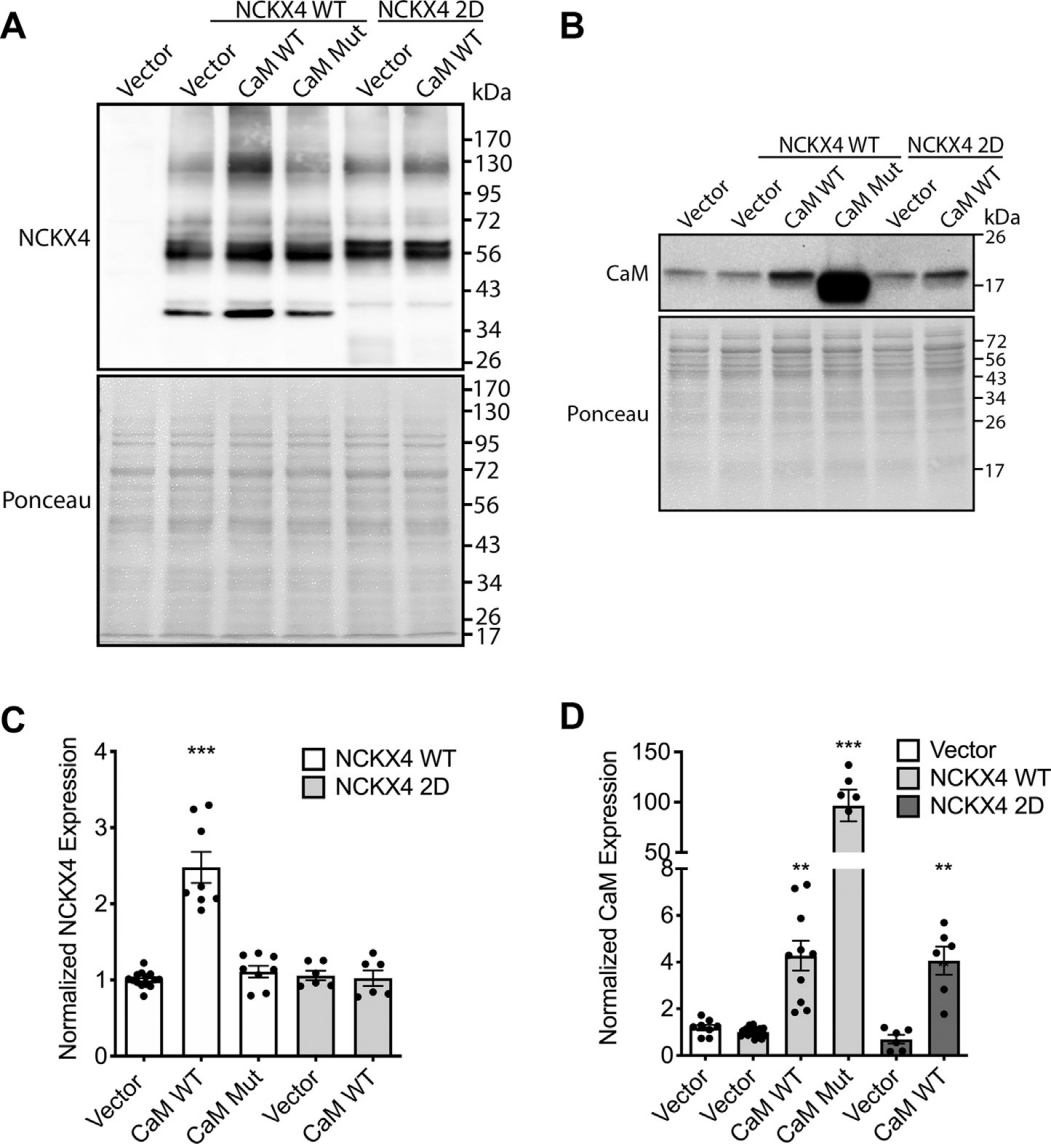
**NCKX4 and CaM expression in transfected HEK293 cells.***A*–*B*, HEK293 cells were transfected with cDNA constructs corresponding to vector-only negative control or either wild-type mouse NCKX4 (NCKX4 WT) or the mouse NCKX4 I328D/F334D double mutant (NCKX4 2D) together with vector (to balance DNA amounts in the transfection cocktail), wild-type CaM (CaM WT), or the Ca^2+^ binding-defective CaM mutant (CaM Mut), as indicated. Twenty microgram (*panel A*) or 60 μg (*panel B*) samples of detergent-solubilized cell extracts were separated by SDS-PAGE and analyzed for protein loading by Ponceau S staining, or for NCKX4 or CaM content by immunoblot with either PSD anti-NCKX4 antibody or rabbit anti-CaM antibody, as noted. The position of molecular mass markers (in kDa) is indicated on the right side of each gel. *C* and *D*, densitometric analysis of the immunoblot data from panel A for NCKX4 (*panel C*), or from the data from panel B for CaM (*panel D*), normalized first for protein loading using densitometry of the Ponceau stain, and then to the average level of expression seen for wild-type NCKX4 plus vector. Data for NCKX4 are summarized from 14, 8, 8, 6, and 6 samples and for CaM from 8, 16, 10, 8, 6, and 6 samples, in the columns from left to right, respectively. Analysis by ANOVA and the Kruskal–Wallis test resulted in F values of 38.9 and 41.6 for NCKX4 and CaM quantification, respectively, with *p* < 0.001 in each case. Samples statistically different from the vector control are indicated (∗∗*p* < 0.01; ∗∗∗*p* < 0.001).

**Figure 7:**
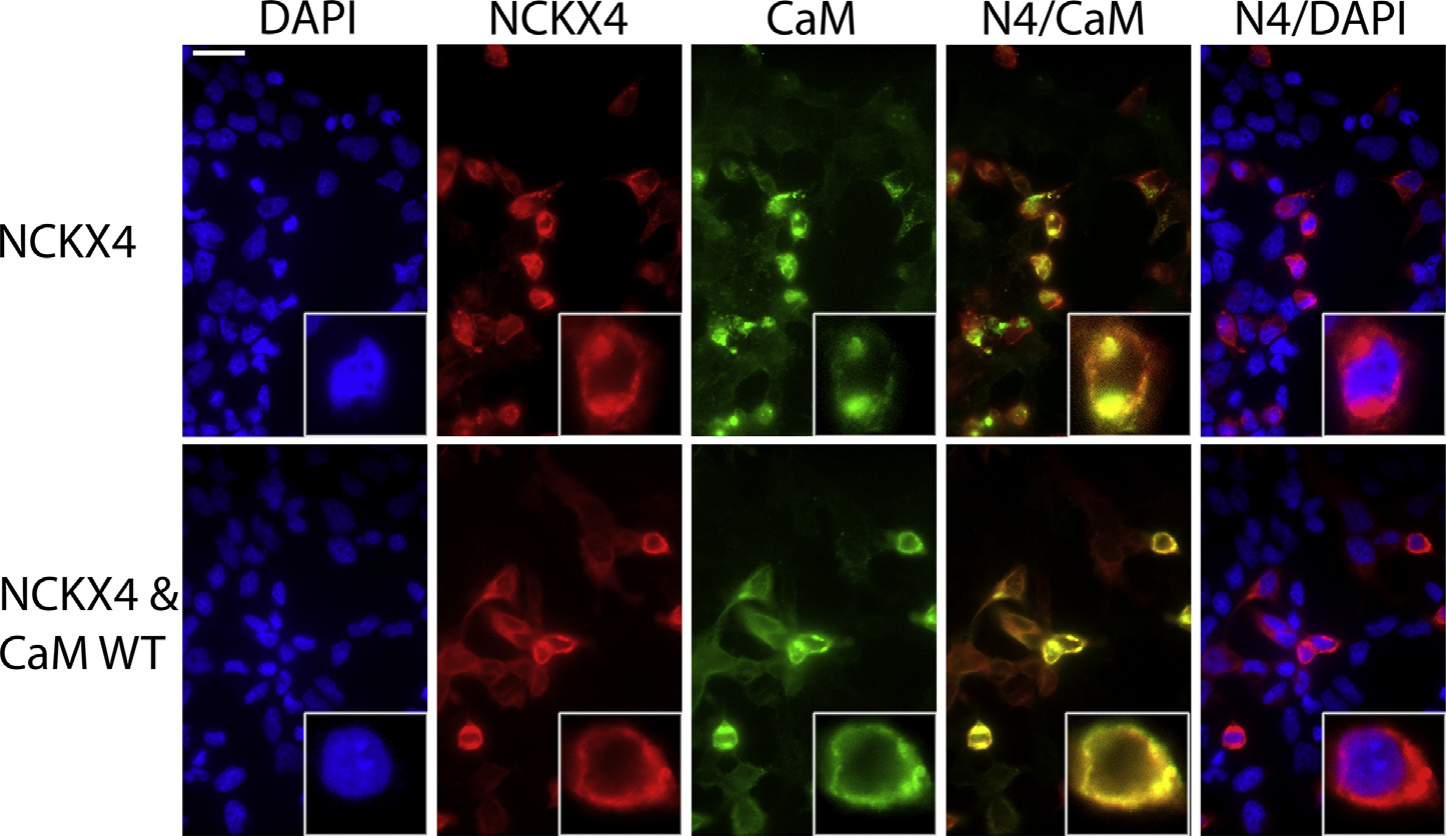
**CaM and NCKX4 localization in cotransfected HEK293 cells.** HEK293 cells grown on coverslips were transfected with cDNA constructs encoding mouse NCKX4 plus either vector control or wild-type CaM. Fixed and permeabilized cells were stained for NCKX4 expression using PSD rabbit anti-NCKX4 and for CaM expression using mouse anti-CaM antibodies; cell nuclei were visualized with DAPI. Merged images of NCKX4 expression together with either CaM (N4/CaM) or DAPI (N4/DAPI) are also illustrated. Photographs for each stain were obtained using the same exposure settings and have been identically adjusted in Adobe Photoshop. Scale bar (*top left panel*) corresponds to 25 μm. The insets at bottom right of each panel are a fourfold magnified view of an individual cell from the field. This figure is representative of more than four independent experiments.

When wild-type CaM was transfected together with wild-type NCKX4 in HEK293 cells, overall CaM expression increased approximately fourfold over the endogenous levels (Fig. 6, *B* and *D*), while NCKX4 expression increased approximately 2.5-fold compared to when transfected alone (Fig. 6, *A* and *C*). Expression of CaM in which a critical aspartic acid residue within each of the four EF-hand domains has been mutated to alanine to render the protein unable to bind Ca^2+^ (30, 31) resulted in a dramatic approximately 100-fold increase in overall CaM expression (Fig. 6, *B* and *D*), but did not influence the level of NCKX4 expression (Fig. 6, *A* and *C*). Note that this anomalously high expression of the CaM mutant is not dependent on NCKX4 coexpression (Fig. S3*A*). Additionally, when the CaM binding-deficient NCKX4 I328D/F334D double mutant was expressed together with CaM, there was no increase in NCKX4 expression (Fig. 6, *A* and *C*), although CaM was again increased about fourfold (Fig. 6, *B* and *D*). These data are consistent with a CaM–NCKX4 interaction that depended upon the region of the NCKX4 cytosolic loop encompassing I328 and F334, as well as Ca^2+^ binding to CaM. As illustrated in Figure 7, endogenous CaM in HEK293 cells appears to cluster at sites of NCKX4 expression, and when cotransfected, CaM and NCKX4 are mostly coexpressed in the same cells. However, although NCKX4 abundance measured by immunoblot was increased by CaM coexpression (Fig. 6), there was no obvious redistribution of the NCKX4 protein observed by immunofluorescence (Fig. 7).

**Figure 8:**
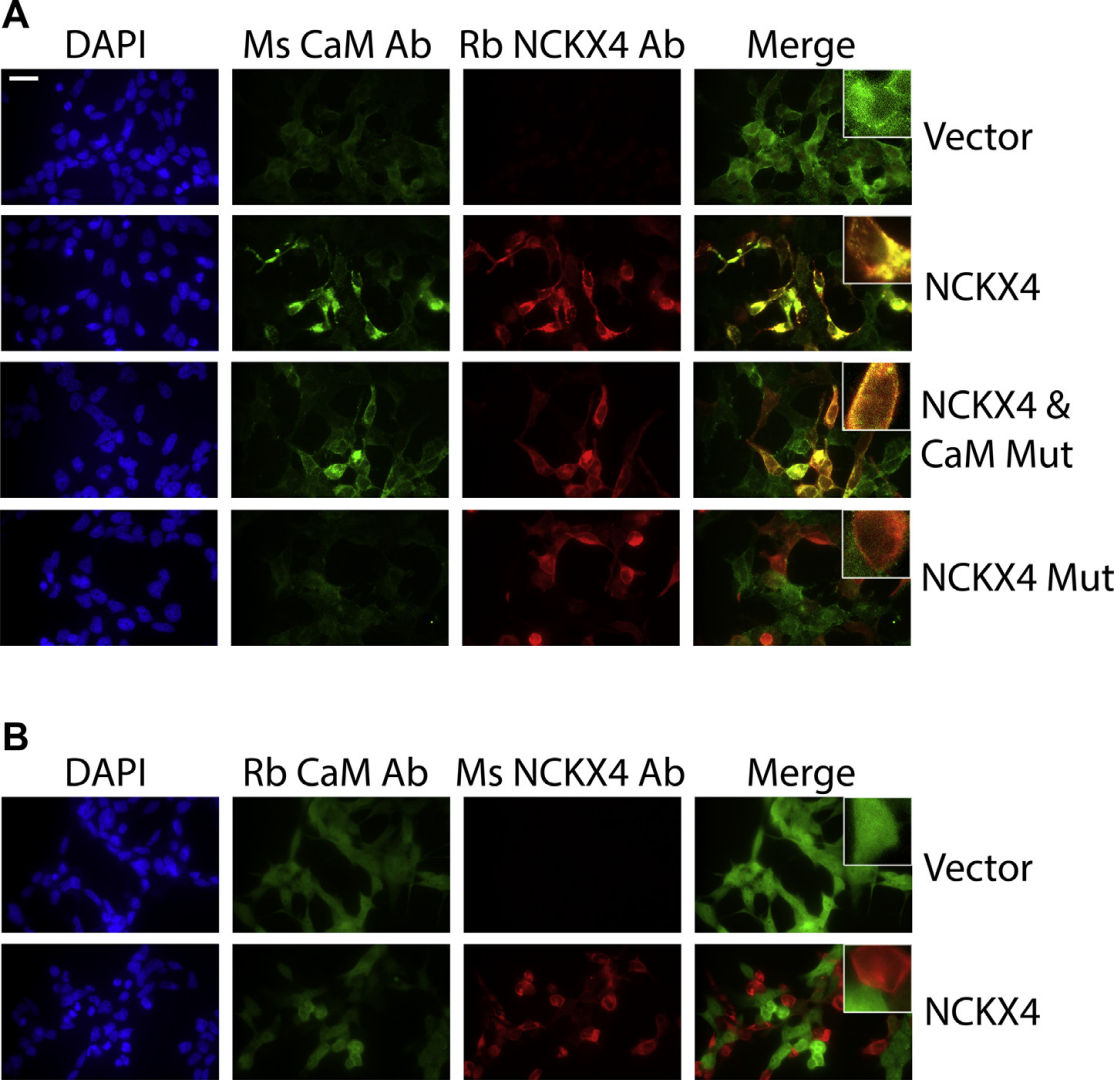
**Relocalization of CaM in NCKX4 transfected HEK293 cells.** HEK293 cells grown on coverslips were transfected with cDNA constructs, as indicated for each row at right: vector-only; NCKX4; NCKX4 and the Ca^2+^ binding-defective CaM mutant (CaM Mut); NCKX4 I328D/F334D double mutant (NCKX4 Mut). Fixed and permeabilized cells were subsequently stained with a combination of either: *A*, mouse anti-CaM and rabbit PSD anti-NCKX4 antibodies, Ms CaM Ab and Rb NCKX4 Ab, respectively; *B*, rabbit anti-CaM and mouse N4MAb anti-NCKX4 antibodies, Rb CaM Ab and Ms NCKX4 Ab, respectively, as noted above each panel. DAPI stain is shown to visualize the nuclei of all the cells within the field of view. All photographs taken for cells stained with the same antibody were captured using identical exposure settings. Contrast and brightness adjustments made in Adobe Photoshop were applied identically to all images for the same antibody. The merged images were further adjusted so overlap (seen in *yellow*) between the staining patterns could be appreciated more readily. Scale bar (top left panel) corresponds to 25 μm. The insets at top right of the merged image panels correspond to a fourfold magnified view of one cell from the field of view. The images in this figure are representative of at least three independent experiments.

To characterize the NCKX4–CaM interaction in more detail, we examined immunofluorescence of transfected HEK293 cells using two different antibodies against each of NCKX4 and CaM. The NCKX4 antibodies were directed against epitopes within the central intracellular loop of NCKX4: i) PSD, a rabbit polyclonal raised against amino acids 294 to 308 (Fig. 1) (13); and ii) N4MAb (a mouse monoclonal generated by the UC Davis/NIH NeuroMab facility [32, 33]), which appeared to recognize an epitope lying mostly within the F2R3 fragment (Fig. 1 and Fig. S1). N4MAb did not recognize the NCKX4 I328D/F334D double mutant, but otherwise recognized wild-type NCKX4 indistinguishably from PSD (Fig. S2). The CaM antibodies used were a mouse monoclonal (from Invitrogen) and a rabbit recombinant monoclonal (from Novus Biologicals). Although both antibodies recognized endogenous and transfected wild-type CaM in HEK293 cells by immunofluorescence, the mouse antibody failed to recognize the Ca^2+^-deficient binding mutant of CaM by immunofluorescence, nor did it recognize CaM on immunoblots (Fig. 8 & Fig. S3), suggesting it recognized a conformationally sensitive epitope.

As illustrated in Figure 8, transfection of wild-type NCKX4 into HEK293 cells resulted in a redistribution of endogenous CaM, when recognized with the mouse antibody (Fig. 8*A*; compare the top two rows of panel A), to sites of NCKX4 expression. This redistribution was not seen with the CaM binding-deficient NCKX4 I328D/F334D double mutant (bottom row of Fig. 8*A*). When the Ca^2+^ binding-deficient CaM mutant was cotransfected with wild-type NCKX4, a CaM staining pattern similar to that observed with just endogenous CaM was observed (center two rows of Fig. 8*A*), suggesting that expression of mutant CaM did not interfere with the normal clustering of endogenous CaM on transfected wild-type NCKX4. The rabbit anti-CaM antibody did not reveal the CaM clustering seen with the mouse one, but instead showed a pattern that generally excluded cells that expressed NCKX4 (Fig. 8*B*). These immunofluorescent data were consistent with the earlier immunoprecipitation data in support of an endogenous interaction between NCKX4 and CaM, supported by basal levels of Ca^2+^ in the cytoplasm. Once bound, CaM adopted a conformation recognized by the mouse antibody but not the rabbit one.

**Figure 9:**
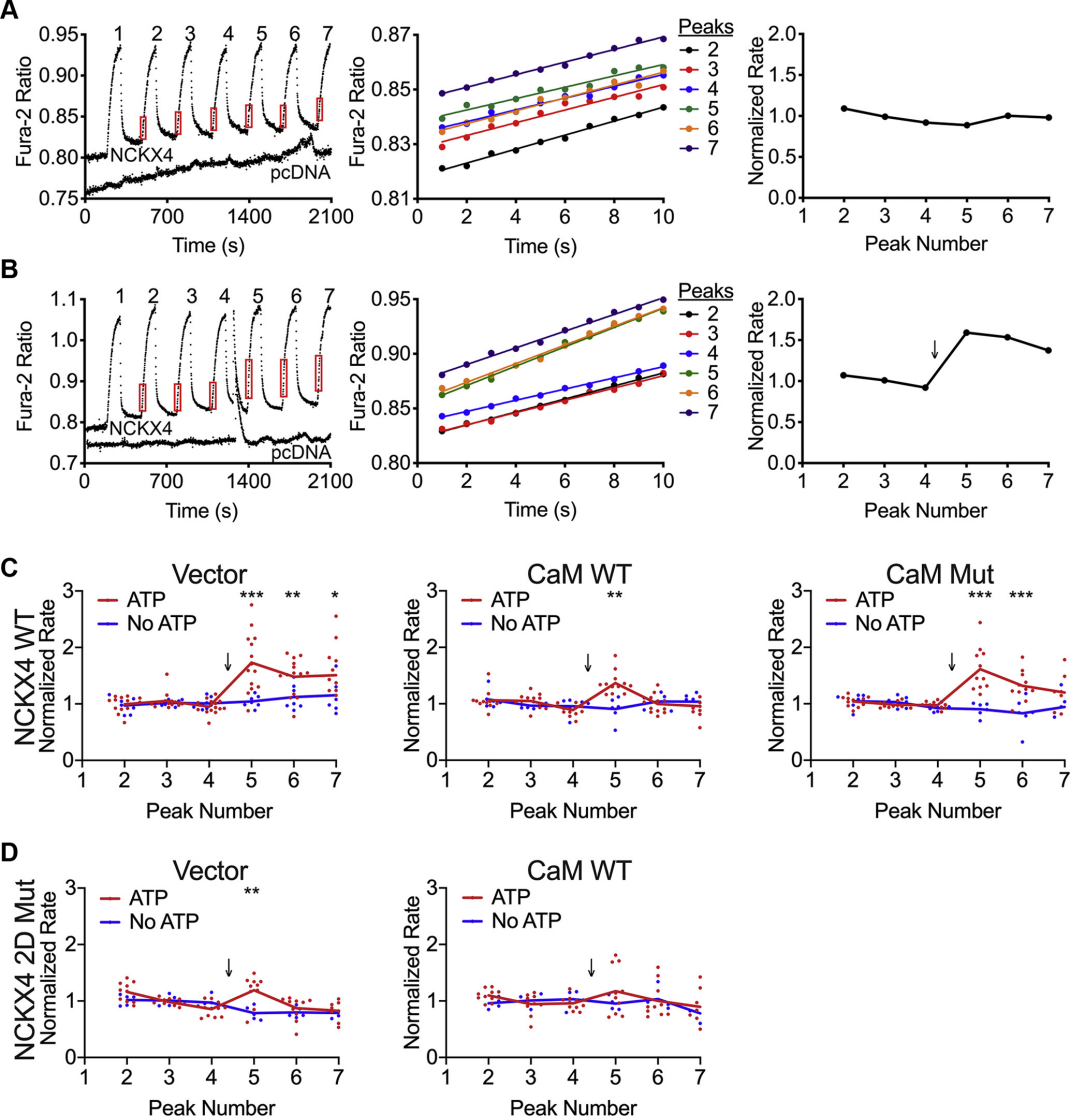
**CaM affects NCKX4 activation by purinergic stimulation.** NCKX activity was measured in transfected, fura-2 loaded, HEK293 cells by alternately perfusing cells with buffers containing 145 mM NaCl, 0 mM KCl, 0.1 mM CaCl_2_, or 145 mM LiCl, 50 μM KCl, 0.1 mM CaCl_2_. Substitution of extracellular NaCl with LiCl induced Ca^2+^ entry *via* NCKX4, and the rate of rise in fura-2 fluorescence ratio was quantified for the steepest portion of each peak induced by the perfusion pulse. The first peak of each series was excluded from analysis because the observed rates were inconsistent with those from subsequent peaks. Normalized rates were determined by comparing to the average rate for peaks 2 to 4. There was no measurable NCKX activity in HEK293 cells transfected with vector, as illustrated in the traces labeled *pcDNA* in the left-hand panels of *A*–*B*. *A*–*B*, representative experiments for mouse NCKX4 or vector (*pcDNA*) transfected cells either untreated (*A*) or stimulated with 0.2 mM ATP (*B*) following perfusion pulse 4. In each case, the left panel shows a representative fura-2 trace, with red boxes indicating the data used for rate calculations for the numbered peaks; the center panel shows expanded and superimposed data from the boxed regions, with the regression line for the data set from each numbered peak; the right panel shows the normalized data from this individual experiment. The *downward arrow* indicates the time of ATP addition. Representative traces for all conditions are compiled in Figure S5. *C*, summary data for HEK293 cells transfected with cDNAs encoding wild-type mouse NCKX4 together with vector (to balance DNA amounts), wild-type CaM (CaM WT), or Ca^2+^ binding-deficient mutant CaM (CaM Mut), and then treated with or without ATP. Data from 7 (vector without ATP), 14 (vector plus ATP), 6 (CaM WT without ATP), 12 (CaM WT plus ATP), 6 (CaM Mut without ATP), and 11 (CaM Mut with ATP) independent experiments are shown. Analysis by two-way ANOVA and the Tukey posttest resulted in F values of 14.7, 8.7, and 6.4 for peak factor, transfection/treatment factor, and their interaction, respectively, with *p* < 0.0001 in each case. Peak rates statistically different between samples treated with and without ATP are indicated (∗*p* < 0.05; ∗∗*p* < 0.01; ∗∗∗*p* < 0.001). Additionally, CaM WT with ATP was significantly different from vector with ATP at peaks 5, 6, and 7 with *p* < 0.001. *D*, summary data for cDNA encoding the mouse NCKX4 I328D/F334D double mutant transfected together with vector or wild-type CaM and treated with or without ATP. Data shown are from 5 (vector without ATP), 10 (vector with ATP), 4 (CaM WT without ATP), and 10 (CaM WT with ATP) independent experiments. Analysis by two-way ANOVA and the Tukey posttest resulted in F and p values of 5.3 and 0.0037, 1.2 and 0.34, and 2.0 and 0.019 for peak factor, transfection/treatment factor, and their interaction, respectively. The only statistically different rates were those of samples treated with and without ATP for peak 5 of the NCKX4 mutant transfected with vector (∗∗*p* < 0.01).

To test the functional consequences of the NCKX4–CaM interaction, we used quantitative fura-2 fluorescent Ca^2+^ imaging in transfected HEK293 cells, as we have previously described (22, 29). This assay measures the rate of Ca^2+^ entry mediated by NCKX4—so-called reverse-mode exchange—when the plasma membrane Na^+^ gradient is reversed by replacement of extracellular Na^+^ with Li^+^. The reproducible and quantitative nature of these measurements is illustrated in Figure 9*A*, where repeated Li^+^ perfusion pulses elicit similar rates of Ca^2+^ uptake and hence increases in fura-2 fluorescence, over time. Note that, as we have observed previously (22, 29), the rate of Ca^2+^ uptake obtained from peak 1 was not consistent with subsequent peaks and so was excluded from further analysis. A comparison of absolute rates of Ca^2+^ transport between wild-type NCKX4, the CaM binding-deficient NCKX4 I328D/F334D double mutant, or either NCKX4 construct together with CaM, did not reveal any obvious and consistent difference in activity (Fig. S4).

These data were surprising, since coexpression of CaM with NCKX4 induced an approximately 2.5-fold increase in NCKX4 protein abundance, as noted above (Fig. 6). To investigate the relation between NCKX4 expression and Ca^2+^ transport activity, we transfected HEK293 cells with different concentrations of DNA and compared protein abundance with activity (Fig. S4). Although we titrated DNA over an eightfold range, we were only able to observe a 2.3-fold range of NCKX4 expression. Nevertheless, over this limited range we observed a similar, though slightly lower, change in activity (1.6-fold), which provided a reasonable approximation to expectation (Fig. S4). On the other hand, the 2.5-fold increase in NCKX4 abundance induced by CaM coexpression resulted in only 1.1-fold increase in activity. This result suggested that the CaM interaction with NCKX4, as well as increasing protein abundance, inhibited NCKX4 activity either allosterically or due to retention of protein on vesicles within the cell near the membrane, so that we were unable to discern any obvious redistribution by immunofluorescence (Fig. 7).

**Figure 10:**
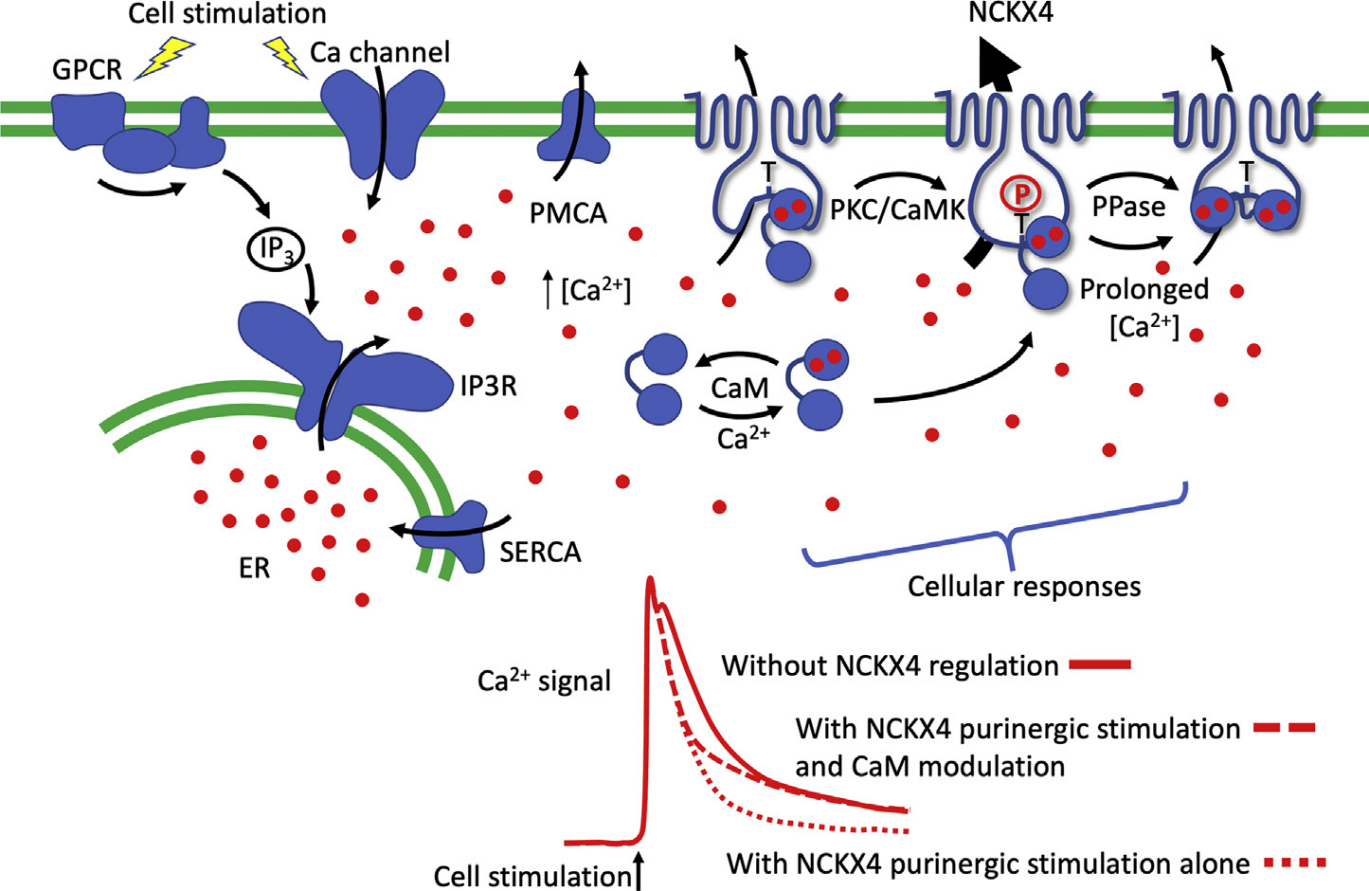
**Schematic illustrating the impact of regulated NCKX4 activity on cellular Ca**^**2+**^**dynamics and responsiveness.** Cellular stimulation, whether by activation of G-protein-coupled receptor (GPCR) signaling, IP_3_ production, and the activation of IP_3_-receptors (IP3R) on the endoplasmic reticulum (ER), or by activation of surface Ca^2+^ permeable channels, leads to an increase in cytosolic [Ca^2+^] (Ca^2+^ is schematically illustrated with *red circles*). As the signal terminates and/or the receptors inactivate, cytosolic Ca^2+^ is lowered by the combined actions of the sarcoplasmic and/or endoplasmic reticulum (SERCA) and plasma membrane (PMCA) Ca^2+^ pumps as well as exchangers such as NCKX4. In many neurons, exchange-driven Ca^2+^ extrusion predominates over other pathways (45, 46). Activation of protein kinase C (PKC) and Ca^2+^-calmodulin-dependent kinase (CaMK) by elevated Ca^2+^ quickly leads to stimulation of NCKX4 activity, which involves phosphorylation of T331, and more rapid Ca^2+^ extrusion. Sustained Ca^2+^ signals lead to full Ca^2+^ loading of NCKX4-bound CaM, which can rearrange on its binding site concomitantly with phosphatase (PPase)-mediated dephosphorylation of T331, reducing the stimulation of NCKX4 activity. These temporal changes lead to shaping of the cell stimulation-induced Ca^2+^ signal and subsequent cellular responses, as illustrated in the lower part of the figure. Without purinergic stimulation of NCKX4, the Ca^2+^ signal shows an initial peak due to entry followed closely by a more slowly developing peak from ER Ca^2+^ release and a subsequent steady decline (*solid red line*). Purinergic stimulation of NCKX4 activity increases the rate of decline of the Ca^2+^ signal (*dotted red line*). Modulation of NCKX4 activity by CaM subsequent to purinergic stimulation leads to an initially rapid decline in Ca^2+^, which subsequently slows as Ca^2+^/CaM reverses the purinergic stimulation of NCKX4 (*dashed red line*).

We have previously reported that NCKX4 activity could be activated by purinergic signaling (22), as illustrated in Figure 9*B*. Here, because rates were determined sequentially within the same experiment, modest differences in activity were readily observed. Using this approach, addition of 0.2 mM ATP to HEK293 cells transfected with wild-type NCKX4 induced a 1.5-fold increase in activity (Fig. 9, *B*–*C*). When wild-type CaM was cotransfected with NCKX4, the ATP-induced stimulation of NCKX4 activity was much smaller and was statistically significantly reduced, compared to vector cotransfection (Fig. 9*C* and Fig. S5). This reduction in activation was more pronounced for peaks 6 and 7, compared to 5, suggesting a time dependence to this phenomenon. Cotransfection of the Ca^2+^ binding-deficient CaM mutant together with NCKX4 resulted in an ATP response that was not significantly different from the response with vector cotransfection (Fig. 9*C* and Fig. S5), in keeping with the observations that coexpression of the CaM mutant does not seem to influence the endogenous CaM–NCKX4 interaction observed by immunofluorescence (Fig. 8*A*). Interestingly, ATP stimulation of exchange activity of the NCKX4 I328D/F334D double mutant resulted in a much smaller increase in activity, significant only for peak 5 when this NCKX4 mutant was expressed alone (Fig. 9*D* and Fig. S5). These observations suggest that the mutations introduced into NCKX4 to abolish CaM binding may interfere independently with purinergic stimulation. Coexpression of wild-type CaM with the NCKX4 double mutant had no statistically significant effect (Fig. 9*D* and Fig. S5), as anticipated from the lack of interaction between these proteins observed by either immunoprecipitation (Fig. 5) or immunofluorescence (Fig. 8). In summary, Ca^2+^/CaM binding to the central cytosolic loop of NCKX4 seems to suppress purinergic activation of the exchanger in a time-dependent manner (see schematic in Fig. 10).

## Discussion

In this article, we used a yeast two-hybrid screening strategy to identify CaM as a binding partner for NCKX4 and demonstrated that even under basal conditions, CaM was bound to NCKX4 in a manner that depended upon Ca^2+^. Cotransfection of wild-type, but not Ca^2+^ binding-deficient, CaM increased NCKX4 protein expression, inhibited transport activity, and suppressed the purinergic stimulation of NCKX4 activity in a time-dependent manner.

While Ca^2+^/CaM is a well-recognized regulator of various cell surface Ca^2+^ channels (34) and the plasma membrane Ca^2+^-ATPase pump (35), few studies have examined its influence on the function of members of the NCX or NCKX Na^+^/Ca^2+^ exchanger families. When NCX1 was originally cloned, the sequence revealed a short cationic and amphipathic region, reminiscent of a canonical CaM binding motif, near the N-terminus of the central cytosolic loop of the protein (36). Further studies by this group revealed that a peptide corresponding to this region had an inhibitory effect on NCX1 activity, leading to the name eXchanger Inhibitory Peptide (XIP). However, they failed to observe either binding of NCX1 to calmodulin or any functional effect of calmodulin on NCX1 activity (37). More recently, reexamination of the NCX1 sequence suggested the existence of a CaM-binding region close to the C-terminus of the NCX1 cytosolic loop (38), a prediction also supported by analysis of the NCX1 sequence through the CaM target database (http://calcium.uhnres.utoronto.ca/ctdb/) (25). Chou *et al*. (38) used recombinant *in vitro* studies to confirm CaM binding to this site and further demonstrated that deletion of the CaM-binding region impeded surface delivery of NCX1 when expressed in HEK293 cells. Coexpression of NCX1, or NCX1 with the CaM-binding site deleted or mutated, together with either wild-type or Ca^2+^ binding-deficient CaM, resulted in complex effects on activity. These data suggest that the CaM interaction with NCX1 may interface with other regulatory regions of the exchanger in a complicated way. Our studies on NCKX4 also revealed a complex interaction between CaM binding, NCKX4 expression and activity, and other regulatory mechanisms that influence NCKX4 activity. However, we did not observe any obvious impact of mutating the NCKX4 CaM-binding region on activity, level of expression, or location of NCKX4 (Figs. 6 and 8).

The function of NCKX4 has been linked to sensory signaling systems, such as visual transduction in cone photoreceptors, and odor detection in olfactory sensory neurons (2, 11, 12). In both cases, elevated Ca^2+^ induced negative feedback on signaling, and NCKX4 acted to modulate this process by extruding Ca^2+^, which resulted in critically important time-dependent adaptation of signal recognition. The precise physiological roles for NCKX4-mediated Ca^2+^ extrusion at other sites of significant expression, such as dental ameloblasts (14, 15), brain neurons, and lung epithelial cells (13), are less clear but may involve similar shaping of signaling processes. Our earlier work (22), as well as the data presented here, suggests that NCKX4 itself is further regulated by the very signaling processes with which it is linked. This complex layering of modulation may be an important way in which sensory signal transduction is tuned in a time-dependent manner. Purinergic signaling is often considered to be an important costimulatory pathway to neurotransmitter signaling in neurons (39). As we demonstrated previously, purinergic signaling results in a rapid stimulation of NCKX4 activity that tends to decline with time (22). Our studies here suggest that Ca^2+^/CaM may provide slowly developing suppression of purinergic stimulation, which helps reset the signaling cascades toward basal levels (see Fig. 10 for a schematic).

A scan of the NCKX4 sequence for CaM-binding motifs (http://calcium.uhnres.utoronto.ca/ctdb/) (25) revealed a strong target in the intracellular loop between transmembrane segments (TMS) 9 and 10 toward the C-terminus of the protein and a modest target within the central cytosolic loop, encompassing amino acids 339 to 353. The latter site overlaps the fragment, amino acids 326 to 345, which we identified as the region required for the CaM–NCKX4 interaction. Although the TMS9–TMS10 binding site was strongly predicted, it lies in a short loop between transmembrane segments, close to the membrane, and with restricted conformational options. These constraints probably prevent CaM from binding to the TMS9–TMS10 site and explain why CaM binding to NCKX4 was abolished—whether measured by biochemical (Fig. 5) or cell biological (Fig. 8) assays—by mutation of I328D and F334D in the cytosolic loop. The partial, but not complete, overlap between the predicted CaM-binding site and the experimentally determined one also suggested a more flexible, longer binding site, which may allow CaM rearrangement on NCKX4 when Ca^2+^ levels rise during a signaling event. This may explain why CaM was bound to NCKX4 under basal conditions, but had a functional effect that developed only slowly following the initiation of purinergic signaling and the rise in cytosolic [Ca^2+^].

The two different CaM antibodies used in this study revealed interesting dynamics associated with CaM binding to NCKX4. Both antibodies recognized endogenous CaM as well as transfected wild-type CaM expressed in HEK293 cells with almost entirely overlapping immunofluorescent patterns of diffuse distribution throughout all cells (Fig. S3). The rabbit antibody recognized the Ca^2+^ binding-deficient CaM mutant, while the mouse antibody did not (Fig. S3). This suggested a conformational preference, so that the rabbit antibody preferred a Ca^2+^-free conformation of CaM, while the mouse antibody preferred a conformation involving or mimicking Ca^2+^ binding. Since it was recognized under basal conditions by both antibodies, wild-type CaM may display conformational flexibility, perhaps biased by antibody binding. Most interestingly, when NCKX4 was expressed in HEK293 cells, the endogenous CaM clustered to the membrane sites where NCKX4 was present and adopted a conformation, which was recognized by the mouse antibody (Figs. 7 and 8). In contrast, not only did the rabbit antibody not recognize this clustered form of CaM, but CaM staining was reduced in cells expressing NCKX4, suggesting a depletion of mobile, cytosolic, Ca^2+^-free CaM upon NCKX4 expression (Fig. 8). It seemed likely that the clustering of CaM represented direct binding to NCKX4, rather than a more generalized redistribution to target sites due to altered Ca^2+^ handling in NCKX4-transfected cells, because the CaM binding-deficient NCKX4 I328D/F334D double mutant, which transports Ca^2+^ as effectively as wild-type, did not result in the redistribution of endogenous CaM (Fig. 8).

Transfection of wild-type CaM into HEK293 cells resulted in a relatively modest increase in CaM protein expression of ∼4-fold over endogenous levels, whereas transfection of the Ca^2+^ binding-deficient CaM resulted in an almost 100-fold increase in expression (Fig. 6). We have no good mechanistic understanding of this unusual phenomenon, which was independent of NCKX4 expression (Fig. S3A). The anomalously high expression of the Ca^2+^ binding-deficient CaM has been previously reported by others (40) using a different expression system than the one employed here. The constructs encoding wild-type and mutant CaM used in this study were identical, excepting the four single-nucleotide mutations introduced to change the four key Asp residues to Ala, suggesting that the difference in expression is unlikely to be due to differences in transfection or expression of these plasmids. Since the increase in expression induced by transfection with wild-type CaM is relatively modest, but very large for the Ca^2+^ binding-deficient mutant CaM, it is possible that CaM normally exerts homeostatic control that restricts its own expression and/or stability in a manner requiring the Ca^2+^-binding sites in the molecule.

Interestingly, cotransfection of CaM together with NCKX4 induced a ∼2.5-fold increase in NCKX4 expression (Fig. 6) but no similarly large increase in activity (Fig. S4). The increase in expression was not seen with the Ca^2+^ binding-deficient CaM mutant, nor with the CaM binding-deficient NCKX4 I328D/F334D double mutant, indicating that physical interaction between CaM and NCKX4 was necessary to observe the increase in protein abundance (Fig. 6). Thus, CaM likely did not act in a general way to increase mRNA or protein synthesis from the plasmid CMV promoter or to alter membrane trafficking events, but presumably increased the stability of the mature NCKX4 protein, reducing its turnover. While NCKX4 protein abundance increased, we did not observe any comparable increase in activity (Fig. S4). Since we also did not observe any obvious subcellular redistribution of NCKX4 induced by CaM (Fig. 7), this suggested that NCKX4 activity was inhibited by the interaction with CaM. Our data did not allow us to distinguish if this effect was allosteric, or if NCKX4 bound to CaM was preferentially retained on vesicles inside the cell (presumably sufficiently close to the membrane to explain the lack of redistribution observed by immunofluorescence).

An alternative explanation also needs to be considered, however. Our activity measurements use the rate of change of the fura-2 fluorescent ratio signal. While this is highly reproducible and quantifiable within an individual experiment, we found these rates to be variable between experiments due to a multiplicity of factors, including the number of cells in a field of view, the transfection efficiency, the fura-2 loading, and the exposure settings in fluorescent acquisition software, which all vary between experiments. It is therefore possible that we simply did not capture rate differences that existed between different samples.

Our previous analysis of purinergic stimulation of NCKX4 identified a requirement for dual activation of both PKC and CaMKII in order to observe the increase in NCKX4 activity. We also showed that mutation of Thr331 to Ala (amino acid coordinates for the longer alternatively spliced isoform; Fig. 1)—one of three potential PKC phosphorylation sites on NCKX4—significantly reduced, although it did not fully abolish, purinergic stimulation (22). T331 lies within the CaM-binding site identified here, amino acids 326 to 345, and is flanked by the I328D and F334D mutants, which together prevent CaM binding. The overlap of these sites may explain why CaM binding reduced the effectiveness of purinergic stimulation and why the NCKX4 I328D/F334D double mutant was also much less responsive to purinergic stimulation.

We examined the nature of purinergic stimulation of NCKX4 using various agonists in our previous publication (22) and concluded that a receptor in the P2Y family was involved that activated a pathway involving phospholipase C, PKC, and CaMKII. In those experiments and the ones reported here, we could not resolve whether the stimulation of NCKX4 transport function involved an intrinsic increase in transport rate, or if the number of transporting exchangers at the surface membrane was increased. Although our data using immunofluorescence of NCKX4 do not support an obvious redistribution of the protein under different conditions, as noted above these experiments could not resolve a movement between closely apposed compartments such as the plasma membrane and juxta-membrane vesicles. Further, more complex, experiments will be required to resolve this issue.

In summary, our data suggest a possible model (see Fig. 10 for a schematic version) where CaM, with Ca^2+^ preloaded on sites 1 and 2 under basal conditions, binds to NCKX4. Alternatively, the CaM–NCKX4 interaction may induce a CaM conformation resulting in cooperative binding of Ca^2+^ to these sites under basal conditions. The CaM interaction stabilizes NCKX4, resulting in an increase in the level of protein abundance, but an inhibition of activity. A purinergic signal-induced increase in Ca^2+^ results in rapid activation of NCKX4 involving PKC and CaMKII activation and phosphorylation of NCKX4 on T331. Subsequently, a slower transition occurs involving Ca^2+^ binding to sites 3 and 4 of NCKX4-bound CaM and a repositioning of CaM at its binding site on NCKX4. This reorientation of CaM on NCKX4 interferes with phosphorylation of T331 and together with phosphatase activity reduces purinergic stimulation of NCKX4 transport and hence acts in a competitive way as a time-dependent brake on this signaling axis. The antagonistic nature of the Ca^2+^/CaM *versus* purinergic axes, and their opposing influence on NCKX4 activity, could help shape the nature of cellular Ca^2+^ signaling events, encouraging the rapid decline of a high signal as NCKX4 activity is stimulated, followed by a longer-lasting lower signal as NCKX4 stimulation is suppressed (Fig. 10). Future experiments will test aspects of this model.

## Experimental procedures

### cDNA constructs

The mouse NCKX4 cDNA construct used for these studies has been previously described (22). This cDNA encodes the full-length 603 amino acid NCKX4 protein that lacks a 19 amino acid sequence (275–283) encoded by an alternatively spliced exon and is the predominant isoform expressed in mouse brain (10). As noted in Figure 1, the amino acid coordinates used in this publication correspond to the NCKX4 protein that includes the additional 19 amino acids. For expression in HEK293 cells, mouse NCKX4 in pcDNA3.1(+) was used. In some experiments, a triple-HA tag (encoding the amino acid sequence YPYDVPDYAYPYDVPDYAYPYDVPDYA) was added recombinantly to the C-terminus of the NCKX4 protein. Plasmid DNA was purified for transfection using Qiagen kits.

For the yeast two-hybrid screen, a DNA fragment encoding the full cytoplasmic loop (minus the alternatively spliced region) was cloned into either the pLexA-C or pLexA-N bait plasmids from Dualsystems Biotech Inc. Primers corresponding to the sites labeled F1 and R1 in Figure 1 contained in-frame EcoRI and SalI restriction sites, respectively, for cloning in pLexA-C, or SacII and SalI, respectively, for cloning into pLexA-N. All subsequent constructs used to define the CaM-binding site within the NCKX4 cytoplasmic loop used the pLexA-C vector, with primers as noted in Figure 1. All F primers had an in-frame EcoRI site and encoded a Met residue to define the N-terminal end of the fragment, while all R primers incorporated a SalI site.

For the GST-binding and pull-down assays, the various EcoRI–SalI fragments were excised from pLexA-C and introduced into pGEX-4T-1 which maintained the same reading frame as pLexA-C. Note that in the pLexA-C vector, the NCKX4 fragments lie N-terminal to the LexA protein fusion partner, but for the pGEX-4T-1 vector, they lie C-terminal to the GST protein fusion partner.

Individual and double amino acid mutations were introduced using an appropriately mismatched primer and the Quikchange II kit (Agilent). All cloned fragments were confirmed by Sanger sequencing performed at the University of Calgary Core DNA Services Laboratory.

The calmodulin expression constructs correspond to chicken CALM2 in the pcDNA3.1(+) vector (provided by Dr Wayne Chen; University of Calgary). The encoded wild-type protein is identical to calmodulin of mouse or human origin. The Ca^2+^ binding-deficient mutant is one in which key aspartic acids in each EF-hand domain (D21, D57, D94, and D130; numbering includes the initiator methionine residue) have all been mutated to alanine (30, 31).

### Yeast two-hybrid interaction screen

The screen was performed using the DUALhybrid kit essentially according to the instructions of the vendor (Dualsystems Biotech) and established methodologies (23, 24). In brief, the two bait constructs described above (one with an N-LexA-NCKX4(F1R1)-C and one with an N-NCKX4(F1R1)-LexA-C bait fusion protein orientation) were each transformed into yeast strain NMY51 and tested for expression and lack of self-activation. A mouse brain cDNA library in the pGAD vector (Dualsystems Biotech) was then transformed into each of these bait strains and plated onto selective media (SD-Trp-Leu-His). Positive colonies were picked, rescreened on nonselective (SD-Trp-Leu) and selective (SD-Trp-Leu-His) plates, and the dependence of growth on the bait plasmid tested. Prey plasmids were isolated from the clones that survived these tests and retransformed into *Escherichia coli* DH5a cells. Positive colonies were picked, plasmid DNA isolated, and the prey inserts sequenced at the University of Calgary Core DNA Services Laboratory.

Yeast two-hybrid growth was also used to define the region of the bait necessary for the interaction with prey. Different bait constructs were transformed together with prey plasmid into NMY51 cells, and growth was assessed using tenfold serial dilutions spotted in grids onto either selective (SD-Trp-Leu-His) or nonselective (SD-Trp-Leu) plates.

### Glutathione-S-transferase fusion protein pull-down tests

GST pull-downs were performed essentially according to established protocols (26, 27). Plasmids encoding different GST-NCKX4 fusion proteins in the pGEX-4T-1 vector were transformed into BL21(DE3) *E. coli* cells for protein expression. Overnight cultures were diluted 1:100 into 50 ml of LB medium, grown for 2 to 3 h at 37 °C with vigorous shaking to an OD of ∼0.5, and then induced with 0.1 mM IPTG for 3 h. Cells were then harvested by centrifugation at 2000*g* for 10 min and resuspended in 1 ml of lysis buffer (150 mM NaCl, 1 M urea, 5 mM DTT, 1 mM EDTA, 25 mM Na-phosphate, pH7.4, containing 17 μg/ml phenylmethylsulfonyl fluoride (PMSF), Roche cOmplete ULTRA Protease Inhibitor cocktail (Sigma-Aldrich Cat# 5892791001; 1 tablet dissolved in 50 ml of buffer), and 100 μg/ml lysozyme). All subsequent procedures were done on ice or in the cold room at 4 °C. Following incubation for 30 min, the cells were sonicated for three sequential cycles using a Branson Sonifier 450 at 50% power and 50% duty cycle for 5 min. The resulting homogenate was centrifuged at maximal speed in a microcentrifuge for 10 min, and the supernatant incubated with 100 μl of Glutathione Sepharose beads (VWR; GE Healthcare) for 30 min with rotation. The beads were then washed three times with 1% Triton X-100, 150 mM NaCl, 1 M urea, 5 mM DTT, 50 mM TrisCl, pH7.5, containing PMSF and protease inhibitors, as above, by centrifugation at 1000*g* for 1 min, and suspended in the wash buffer as a 10% slurry. 100 μl of this slurry was then incubated with 1 μg of purified CaM (provided by Dr Hans Vogel; University of Calgary) in the presence or absence of 0.1 mM CaCl_2_ for 30 min with rotation. The beads were washed three times in the same buffer, and the residual supernatant removed by sequential centrifugation and careful pipetting. The beads were then eluted with 15 μl of 20 mM reduced glutathione, 10% glycerol, 50 mM TrisCl, pH 7.5 for 5 min at room temperature. The eluate was mixed with an equal volume of 4X sample buffer, and duplicate 10 μl aliquots were analyzed by SDS-PAGE and immunoblotting for GST and CaM on separate gels. The inclusion of Triton X-100 and urea during this procedure was necessary to improve the solubility of the GST fusion proteins and reduce nonspecific binding of CaM to the immobilized proteins.

### Cell culture and transfections

HEK293 cells were grown in Dulbecco’s modified Eagle’s medium supplemented with 10% fetal bovine serum, 1% nonessential amino acids, 4 mM L-glutamine, and 1% penicillin/streptomycin at 37 °C under 5% CO_2_ and transfected at about 50% confluency with Ca^2+^-phosphate precipitation as previously described (22). Cells were used for experiments 2 days following transfection. Cells for microscopy assays were grown on poly-D-lysine-coated coverslips. For other experiments, cells were grown in culture dishes of various sizes. To prepare whole cell, postnuclear, extracts, the cells were rinsed with cold PBS (130 mM NaCl, 3 mM KCl, 0.1 mM CaCl_2_, 0.1 mM MgCl_2_, 8 mM Na_2_HPO_4_, 2 mM KH_2_PO_4_, pH7.2), solubilized in radioimmunoprecipitation assay (RIPA) buffer (1% Triton X-100, 150 mM NaCl, 25 mM TrisCl, 1 mM EDTA, pH7.5) containing 0.5 μg/ml leupeptin and 0.1 mM PMSF, for 15 min on ice followed by centrifugation at 15,000*g* for 10 min, and the supernatant was removed and used as directed for assays. For the experiments of Figure 6, cells were solubilized in a modified RIPA buffer that additionally contained 0.5% sodium deoxycholate, 1% Aprotinin (Sigma, Cat# A6279), Roche cOmplete ULTRA Protease Inhibitor cocktail (1 tablet dissolved in 10 ml of buffer), and 1 mM PMSF. Membrane fractions were prepared as described below.

### Membrane preparation from mouse brain or transfected HEK293 cells

Mice were maintained and treated in accordance with the guidelines of the Canadian Council on Animal Care as determined by the University of Calgary Animal Care Committee. Membrane fractions were isolated from brains of both wild-type and *Nckx4* knockout mice according to published procedures (28). This method was also adapted to prepare membrane fractions from transfected HEK293 cells. In brief, a dissected mouse brain was coarsely chopped and then disrupted by hand in 10 volumes of cold homogenization buffer (250 mM sucrose, 1 mM EDTA, 25 mM TrisCl, pH7.5 containing 0.5 μg/ml leupeptin and 0.1 mM PMSF) using a Dounce homogenizer with 15 strokes with the loose pestle followed by 15 more with the tight pestle. For HEK293 cells, cells were washed with cold PBS, removed from the culture dish by pipetting with homogenization buffer using 2 to 3 ml for a 10 cm dish, and disrupted with a Dounce homogenizer. All subsequent steps were performed either on ice or at 4 °C. The extract was centrifuged at 5000*g* for 5 min. The supernatant was removed and centrifuged at 12,000*g* for 20 min. The supernatant (referred to as S12) was saved frozen in aliquots at −80 °C. For some experiments in which the pellet (referred to as P12) was used directly, the resuspended sample was either frozen in aliquots at −80 °C or dissolved directly in RIPA buffer. For experiments in which further membrane fractionation was used, the P12 pellet was resuspended in 4 ml of 5 mM TrisCl, pH7.5, and diluted with 2.13 volumes of 50% sucrose in 10 mM TrisCl, pH7.5, and placed in the bottom of a Ti70 centrifuge tube. Solutions of 28.5% sucrose and 10% sucrose in 10 mM TrisCl, pH7.5 were carefully layered over the sample, which was centrifuged at 85,000*g* for 30 min. Material at the second interface from the top was carefully pipetted out of the tube, diluted three times with distilled water, and centrifuged at 150,000*g* for 1 h. This pellet (referred to as P150), which was enriched in synaptic markers and NCKX4 protein (13), was resuspended in either RIPA buffer directly or 5 mM TrisCl, pH7.5 and stored frozen in aliquots at −80 °C.

### CaM pull-down tests

Samples were solubilized and diluted to 1 ml with RIPA buffer containing 1 mg/ml ovalbumin. Approximately 300 μl of a 10% slurry of CaM affinity resin beads (Agilent; Cat# 214303) in RIPA buffer plus ovalbumin was added to the samples, with or without 2 mM CaCl_2_ (1 mM Ca^2+^ final free concentration). The samples were then incubated at room temperature for 30 min, followed by washing three times with RIPA buffer, with or without Ca^2+^ as appropriate, by centrifuging at 1000*g* for 1 min, and the residual supernatant was removed by sequential centrifugation and careful pipetting. The samples were then eluted in 15 μl of 4x sample buffer for 5 min at room temperature, and 10 μl taken for analysis by SDS-PAGE and immunoblot.

### Antibody reagents and immunological protocols

The rabbit polyclonal anti-NCKX4 antibody (PSD) was generated against a synthetic peptide corresponding to amino acids 294 to 308 from the cytoplasmic loop of mouse NCKX4, and serum was affinity purified using peptide attached to agarose beads, as previously described (13). The mouse monoclonal anti-NCKX4 antibody, N4MAb (clone N414/25), was generated against a fusion protein containing amino acids 246 to 424 of human NCKX4 by the UC Davis/NIH NeuroMab Facility (neuromab.ucdavis.edu) and was obtained from Antibodies Inc (32, 33). Three different anti-CaM antibodies were used. A mouse monoclonal (from Millipore; Cat# 05-173) was used for immunoblotting in the coimmunoprecipitation experiments of Figures 4 and 5. A mouse monoclonal (from Invitrogen; Cat# MA3-917) and a rabbit recombinant monoclonal (from Novus Biologicals; Cat# NBP2-67413) were used for all other experiments. The antibody against GST was raised in rabbits (41). The rabbit polyclonal antibody against the HA epitope was from Sigma-Aldrich; Cat# H6908. The rabbit polyclonal antibody against actin was from Sigma-Aldrich; Cat# A2668. The rabbit polyclonal antibody against CaMKII was from Cell Signaling Technology; Cat# 3362S.

Samples for immunoblotting were solubilized in (final) 2X sample buffer, treated either at room temperature or at 56 °C for 5 min, separated on 9% (for NCKX4), 12% (for GST), or 15% (for CaM) SDS polyacrylamide gels (42), transferred to nitrocellulose membranes (43), and stained with 0.1% Ponceau-S dye to confirm equal loading. Membranes were blocked with 5% skim milk powder in PBS containing 0.05% Tween-20 and incubated with appropriately diluted antibodies overnight at 4 °C. The membranes were washed in PBS-Tween, incubated with HRP-conjugated goat anti-rabbit or antimouse secondary antibodies (Jackson ImmunoResearch Laboratories) diluted 1:10,000 for 1 h at room temperature, washed, and then visualized by chemiluminescence using Pierce ECL reagents (Thermo-Fisher). For CaM detection following immunoprecipitation with anti-NCKX4 antibodies, the SuperSignal Femto reagent was used (Thermo-Fisher). Images were captured using either an ImageQuant 4000 (GE Healthcare Bio-Sciences) or a ChemiDoc MP Imaging System (Bio-Rad) and quantified following conversion to optical density using ImageJ (NIH) software.

Samples for immunoprecipitation were dissolved in 1 ml of RIPA buffer plus 1 mg/ml ovalbumin by rotation for 5 min at 4 °C, microcentrifuged at maximal speed for 5 min, and the supernatant transferred to a fresh tube. CaCl_2_ was added to some samples (as indicated) to a final total concentration of 2 mM (final free [Ca^2+^] of 1 mM). For brain membrane samples (P150), which lacked appreciable endogenous CaM, 10 μg of purified protein (provided by Dr Hans Vogel; University of Calgary) was added to the solubilized samples. Samples supplemented in this manner were then incubated with 1 μg of rabbit anti-NCKX4 PSD antibody for 1 h at 4 °C with rotation, followed by 100 μl of a 20% slurry of protein-G-Sepharose beads (VWR; GE Healthcare) in RIPA buffer plus ovalbumin, and rotation was continued for 10 min. The beads were then washed by centrifugation at 3000 rpm in a microcentrifuge for 1 min three times with 1 ml of RIPA buffer plus ovalbumin with or without Ca^2+^ according to the original sample composition. The residual supernatant was removed by sequential centrifugation and careful pipetting. The final pellet of beads was then treated with 15 μl of 4X sample buffer and thorough mixing for 5 min at room temperature. 10 μl of eluate was analyzed by immunoblot. Immunoprecipitated samples from brain membranes were eluted using 10 μg of purified antigenic peptide for 5 min at room temperature, supplemented with sample buffer, and analyzed by immunoblot.

Immunofluorescent experiments were conducted on transfected HEK293 cells grown on poly-D-lysine-coated coverslips, as previously described (44). Briefly, 2 days following transfection, cells were washed with PBS, fixed in 3% paraformaldehyde in PBS, permeabilized in 0.2% Triton X-100 in PBS, blocked with 0.2% fish gelatin in PBS, and then incubated with either mouse anti-NCKX4 N4MAb antibody (1:500) and rabbit anti-CaM antibody (1:100) or rabbit anti-NCKX4 PSD antibody (1:50) and mouse anti-CaM antibody (1:100) in PBS with 0.2% gelatin for 2 h at room temperature. Antibody staining was then revealed with Alexa Fluor-488 conjugated donkey antimouse secondary antibody (1:100; Thermo-Fisher) and Cy-3 conjugated donkey anti-rabbit secondary antibody (1:100; Jackson ImmunoResearch Laboratories). In some experiments, Ca^2+^ was omitted from the PBS without any qualitative effect on antibody staining. 4’,6-diamidino-2-phenylindole (DAPI; 1 μg/ml, Thermo-Fisher) was included in the last wash, and the coverslips were mounted in Fluoromount (Sigma), visualized using a Zeiss Axioscope II microscope with a 63X oil objective, and images were captured monochromatically with a Spot Imaging RT digital camera. The images shown in figures have been reduced from 16 to 8-bit, contrast and brightness were adjusted and pseudo-colored, using Adobe Photoshop. When a series of images were compared (*e.g.*, Fig. 8), exposure times used for capture, and any contrast and brightness adjustments, were made identically to all images.

### NCKX4 activity determination by Ca^2+^ imaging

HEK293 cells grown on poly-D-lysine-coated coverslips were used 2 days following transfection. The cells were loaded with 5 μM fura-2-AM (Thermo-Fisher) at room temperature for 40 min, then mounted in a perfusion chamber on the stage of an Olympus IX50 inverted microscope, and observed through a 20x UAPO/340 0.75NA objective. The activity of NCKX4 was then determined essentially as described previously (22). In brief, the cells were alternatively perfused with Na^+^-containing buffer (145 mM NaCl, 10 mM Hepes-tetramethylammonium, pH 7.4, 10 mM D-glucose, and 0.1 mM CaCl_2_) or with buffer in which the Na^+^ had been replaced with 145 mM LiCl and 50 μM KCl. Under these conditions, the membrane Na^+^ gradient was reversed, and so Ca^2+^ entry *via* NCKX4 can be measured as an increase in the fura-2 ratio. A limiting amount of K^+^ was used in the perfusate to reduce the rate of NCKX4 transport, thus allowing quantitative assessment of NCKX4 activity, as described previously (22). This alternating perfusion was repeated seven times in succession. Purinergic stimulation was assessed by including 0.2 mM ATP in the perfusates following the fourth cycle. NCKX4 activity was measured as the rate of increase in fura-2 ratio during the Li^+^-50 μM K^+^ perfusion steps, captured once every second over a field of 20 to 50 cells using excitations at 340 and 380 nm and emission at 510 nm with a QuantEM:5125C EMCCD camera and EasyRatioPro software (Photon technology international, HORIBA Scientific). Because the initial rise in fura-2 ratio was not instantaneous, but gradual over a few seconds, and slightly variable from experiment to experiment due to mixing during the perfusion change, we found it difficult to apply a consistent time offset as a way to choose the data points for rate extraction. Instead, RStudio was used to find the steepest slope among all linear regressions of ten data point intervals during the Li^+^-50 μM K^+^ perfusion step. A few of these selections required manual curation to ensure they did not reflect anomalous changes in ratio due to artifacts. The fura-2 ratio rates were normalized to the average of peaks 2, 3, and 4, because the first peak gave rate data inconsistent with subsequent peaks.

### Statistical analysis

Normalized data were compared using either one-way ANOVA with the Kruskal–Wallis test or two-way ANOVA with the Tukey test using GraphPad Prism software. *p* values <0.05 were considered statistically significant.

## Data availability

All data are either presented in this paper or available from the corresponding author.

## Conflict of interest

The authors declare that they have no conflicts of interest with the contents of this article.
