# Supplementary material for: Calmodulin binds and modulates K+-dependent Na+/Ca2+-exchanger isoform 4, NCKX4
Source: J Biol Chem. 2020 Nov 23;296:100092. doi: 10.1074/jbc.RA120.015037 (PMC7949085; doi:10.1074/jbc.RA120.015037)
Supplement: Figures S1–S5 [file mmc1.pdf]

Supporting Information for

Thibodeau, Yang, Sharma & Lytton

“Calmodulin binds and modulates K^+-dependent Na^+/Ca^{2+}-exchanger isoform 4, NCKX4”

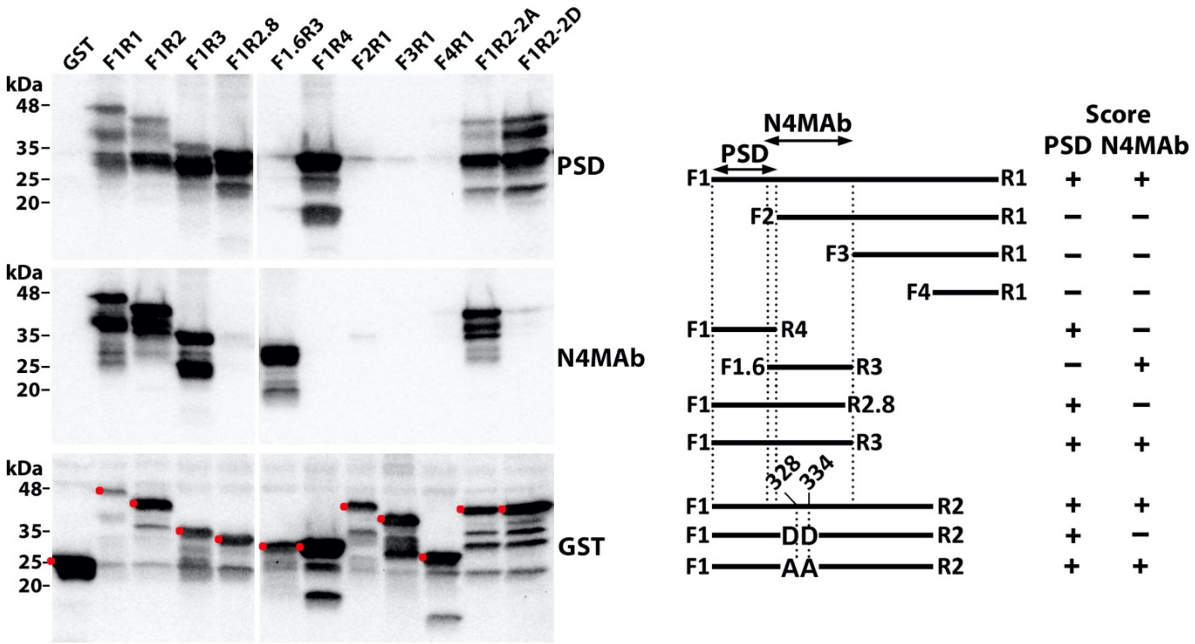

Supplemental Figure 1. Epitope localization of NCKX4 antibodies.
The epitopes for rabbit anti-NCKX4 PSD antibody and the mouse anti-NCKX4 N4MAb antibody were localized using the GST-fusion proteins as described in the legends to Figs. 1 and 4, and *Experimental Procedures*. GST-fusion F1.6R3 contains an additional 9 amino acids at the N-terminal end compared to fragment F2R3, while F1R2.8 has 8 fewer amino acids at the C-terminal end compared to fragment F1R3. Left-hand panels show immunoblots with the indicated antibody. The position of molecular mass markers (in kDa) is noted at left and the position of the full-length GST-fusion proteins is indicated with a red dot. The schematic at right shows the extent of each GST-fusion fragment, with the regions required for a positive antibody interaction noted above, and scored for binding of the antibody indicated at right. Binding of N4MAb to the F1R2 fragment was abolished by mutation of both Ile328 and Phe334 to Asp (F1R2-2D), but not to Ala (F1R2-2A). Note that faint bands observed at about 33 kDa in lanes corresponding to F1.6R3, F2R1, F3R1 and F4R1 in the PSD blot, as well as in the lane corresponding to F2R1 in the N4MAb blot, do not align with the GST-fusion proteins in those lanes, and are therefore likely non-specific.

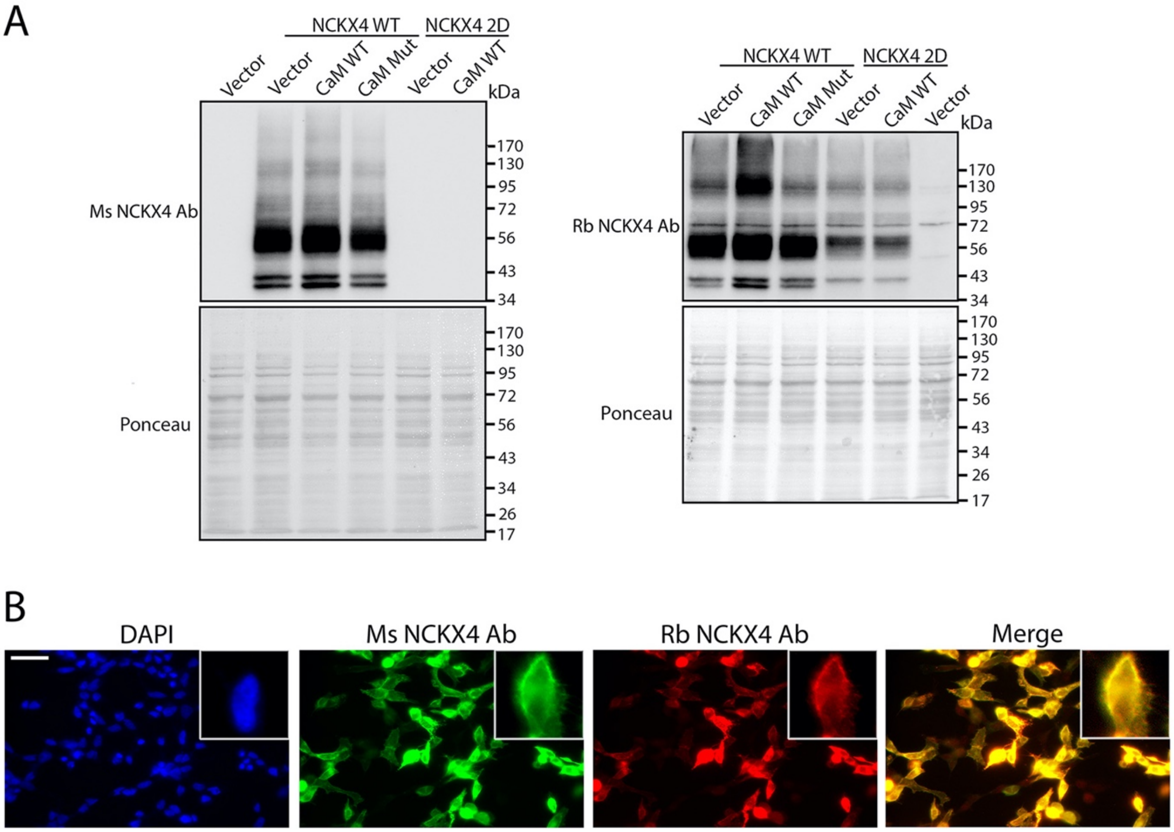

Supplemental Figure 2. Comparison of NCKX4 antibody specificity.
The specificity of the rabbit anti-NCKX4 PSD and the mouse anti-NCKX4 N4MAb antibodies was determined using HEK293 cells transfected with cDNAs encoding vector only (negative control), mouse NCKX4 (NCKX4 WT) or the mouse NCKX4 I328D/F334D double mutant (NCKX4 2D), together with either vector, CaM (CaM WT) or Ca^{2+} binding-deficient mutant CaM (CaM Mut), as indicated.
A. 20 µg samples of detergent-solubilized cell extracts of the indicated samples were separated by SDS-PAGE and analyzed for protein loading by Ponceau S staining, or for NCKX4 content by immunoblot with either the mouse anti-NCKX4 N4MAb or the rabbit anti-NCKX4 PSD antibodies, as noted. The positions of molecular mass size markers (in kDa) are shown at the right side of the gels.
B. HEK293 cells grown on coverslips were transfected with cDNAs encoding NCKX4 and CaM, and analyzed by double label immunofluorescence using mouse N4MAb (Ms) and rabbit PSD (Rb) anti-NCKX4 antibodies. DAPI was used to identify the nuclei of all cells in the field. The scale bar (left panel) corresponds to 25 µm. Insets at the top right of each micrograph are four-fold magnifications of an individual cell in the field.
Note that the mouse anti-NCKX4 antibody recognized wild type NCKX4, but not the NCKX4 I328D/F334D double mutant on immunoblots, while the rabbit anti-NCKX4 antibody recognized both wild type and the double mutant (panel A). Both antibodies displayed essentially identical immunofluorescent patterns for wild type NCKX4 expression (panel B).

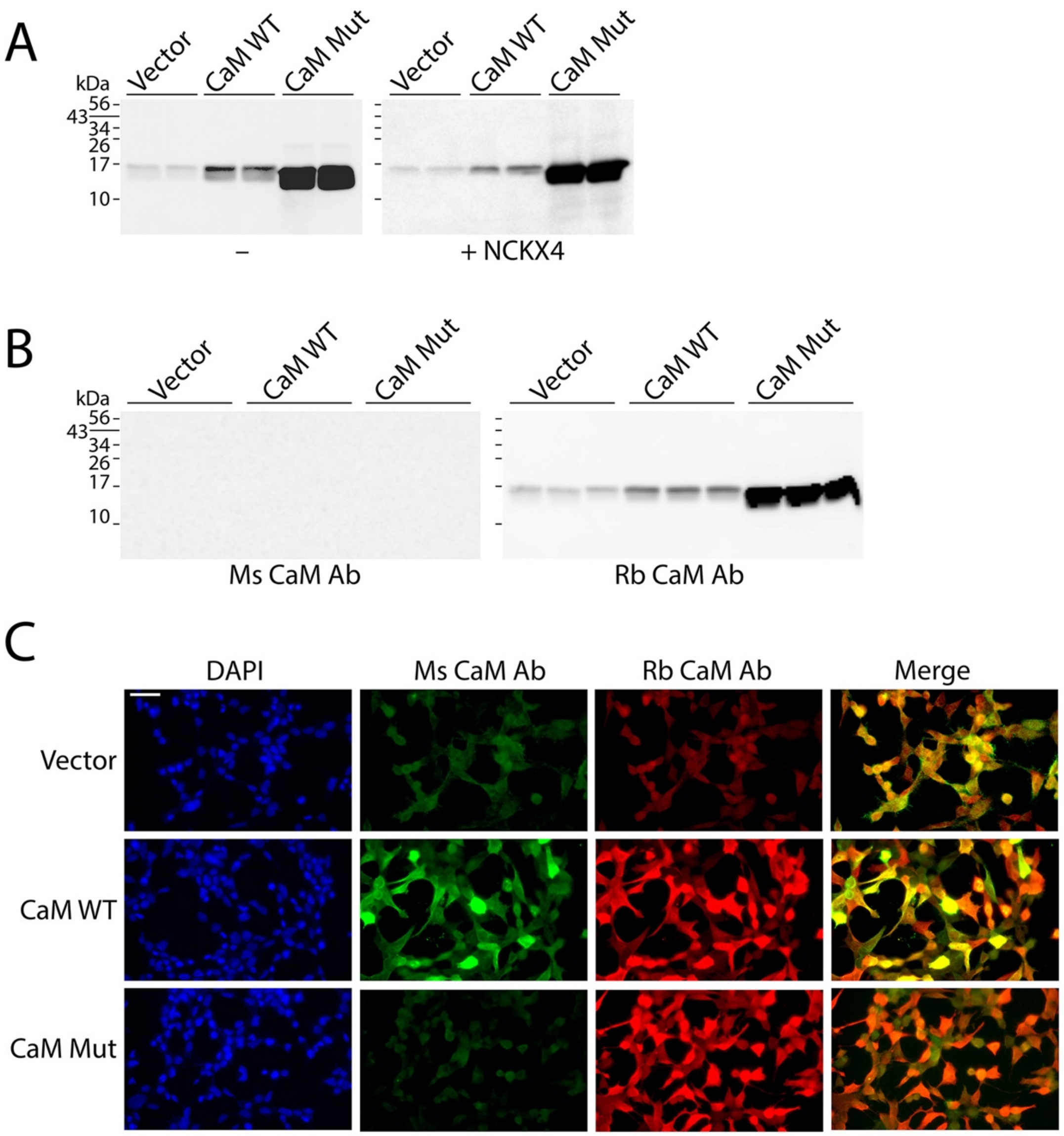

Supplemental Fig. 3. Comparison of CaM expression and antibody specificity.
HEK293 cells were transfected with cDNAs corresponding to either pcDNA control (Vector), wild type CaM (CaM WT) or Ca^{2+} binding-deficient mutant CaM (CaM Mut), as indicated.
A. The level of CaM expression is not influenced by NCKX4 co-expression. cDNAs were transfected either alone (–) or together with NCKX4 cDNA, as indicated. Duplicate samples of post-nuclear cell lysates were separated on 15% SDS gels and then probed with rabbit anti-CaM antibody.
B. The mouse anti-CaM antibody does not recognize CaM separated by SDS-PAGE. Triplicate samples of post-nuclear lysates from cells transfected as indicated were separated on a 15% SDS gel and probed with the mouse ant-CaM antibody (Ms CaM Ab; left blot). The membrane was subsequently stripped and reprobed with rabbit anti-CaM antibody (Rb CaM Ab; right blot).
C. CaM localization was determined using double label immunofluorescence with mouse and rabbit anti-CaM antibodies. HEK293 cells grown on coverslips were transfected as indicated at left. DAPI was used to identify the nuclei of all cells in the field. The scale bar (top left panel) corresponds to 25 µm. Note the extensive overlap in staining between the two antibodies observed with vector (i.e. endogenous CaM) and CaM WT, but not with the CaM Mut, because the mouse anti-CaM antibody does not recognize the CaM Mut.

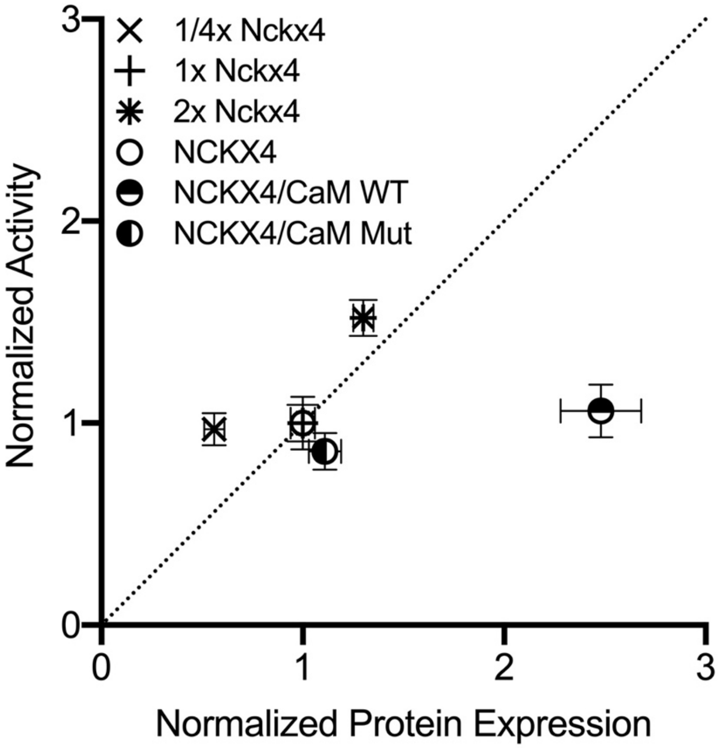

Supplemental Fig. 4. The relation of NCKX4 expression levels to activity measurements.
The relation between NCKX4 expression, determined by immunoblot, and NCKX4 activity, determined by quantitative Ca^{2+} imaging, is shown for two sets of transfected HEK293 cells.
In the first set, shown as *circles*, cells were co-transfected with NCKX4 alone (with pcDNA to balance DNA amounts), CaM wild type (WT), or the Ca^{2+} binding-deficient CaM mutant (Mut). In the second set, shown as *crossed-line* symbols, Nckx4 cDNA was transfected alone in varying amounts of DNA (1/4x normal, 1x normal or 2 x normal). Note that the 1x normal titration and NCKX4 alone data points overlap. For each data set, both immunoblot and activity measurement have been normalized to those observed for Nckx4 transfected alone in the normal amount. Data are shown as mean values ± SEM from 4 (immunoblot from DNA titration), 6 (activity from DNA titration), 8–14 (immunoblot from CaM co-expression), or 17–21 (activity from CaM co-expression) independent experiments. In some instances, the error bars are obscured by the data point symbol. The dotted line along the diagonal illustrates the anticipated linear relation between protein expression and activity.

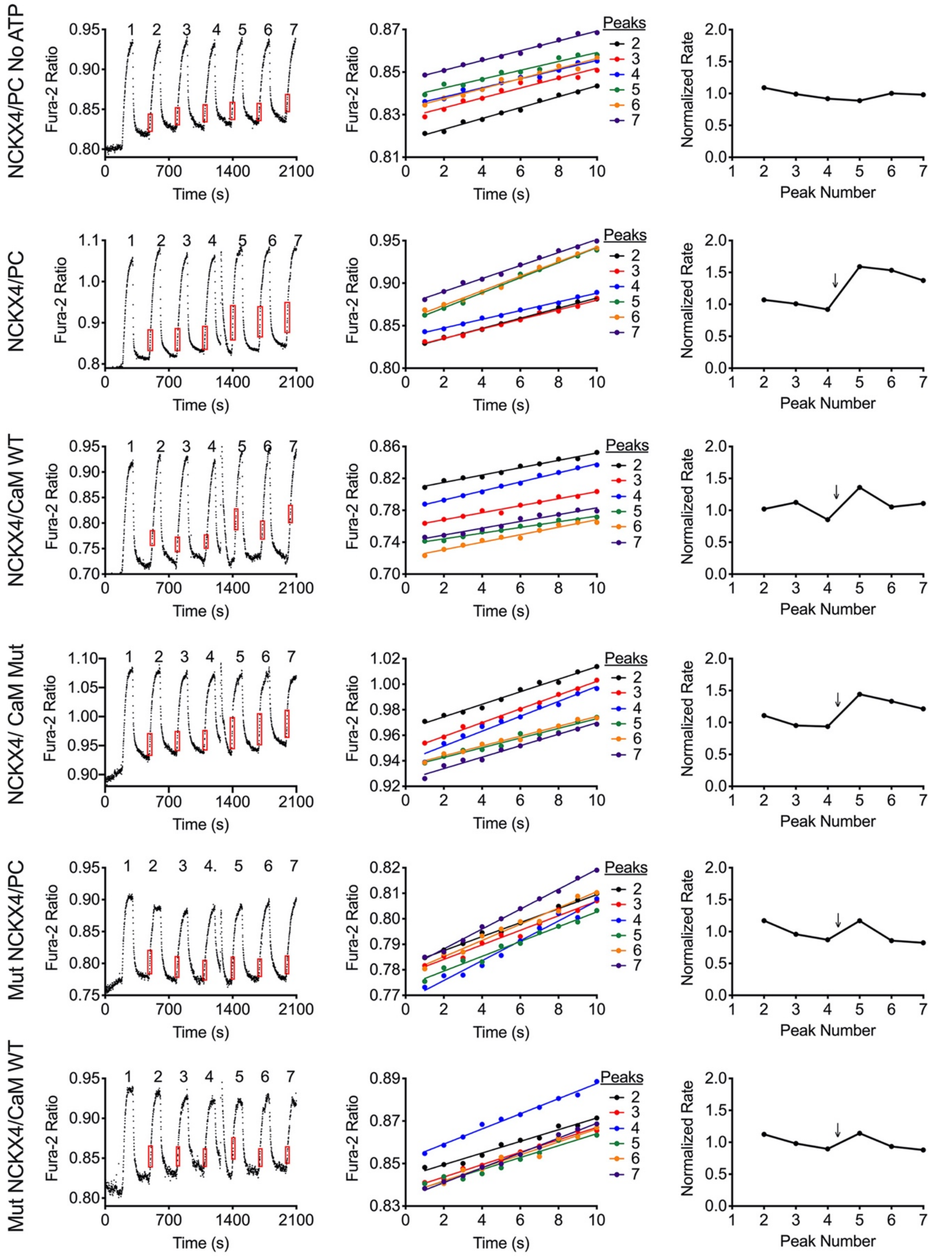

Supplemental Fig. 5. Exemplar traces for NCKX4 activity measurements.
NCKX activity was measured in transfected, fura-2 loaded, HEK293 cells as described in the legend to Fig. 9 and *Experimental Procedures*. Representative experiments are shown for each transfection

Continued…

combination indicated at left: mouse NCKX4 plus pcDNA vector (to balance DNA amounts; NCKX4/PC), NCKX4 plus CaM wild type (NCKX4/CaM WT), mouse NCKX4 plus Ca^{2+} binding-deficient mutant CaM, (NCKX4/CaM Mut), and mouse NCKX4 I328D/F334D double mutant transfected with pcDNA vector (Mut NCKX4/PC) or with wild type CaM (Mut NCKX4/CaM WT). 0.2 mM ATP was added to each experiment except that of the top row at the time noted by the *downward arrow.* In each row, the left panel shows a representative fura-2 trace, with *red boxes* indicating the data used for rate calculations for the numbered peaks; the center panel shows expanded and superimposed data from the boxed regions, with the regression line for the dataset from each numbered peak; the right panel shows the rates, normalized to the average rates from peaks 2 to 4, from that individual experiment. The top two rows are identical to those shown in Fig. 9.
